# Direct Exposure to Mass Shootings Among US Adults

**DOI:** 10.1001/jamanetworkopen.2025.0283

**Published:** 2025-03-07

**Authors:** David C. Pyrooz, James A. Densley, Jillian K. Peterson

**Affiliations:** 1 Department of Sociology and Institute of Behavioral Science, University of Colorado Boulder, Boulder; 2 School of Criminology and Criminal Justice, Metropolitan State University, St Paul, Minnesota; 3 The Violence Prevention Project Research Center, Hamline University, St Paul, Minnesota

## Abstract

**Introduction:**

Mass shootings, defined as incidents where 4 or more people are shot with a firearm, have become a significant public health concern in the US.

**Objective:**

To estimate the prevalence of direct exposure to mass shootings among US adults and identify the sociodemographic groups most affected.

**Design, Setting, and Participants:**

A survey was administered in January 2024 to a sample of 10 000 respondents (18 years or older) designed to be representative of US adults using a multistage matched sampling design. In addition to exposure to mass shootings, the survey also collected sociodemographic information, including age, gender, self-reported race and ethnicity, and socioeconomic status.

**Exposure:**

Survey respondents were asked about their lifetime presence at the scene of a mass shooting, any physical injuries sustained (including being shot or trampled or experiencing a related physical injury), and the features of such incidents.

**Main Outcome and Measures:**

Multivariable logistic regression was used to generate adjusted odds ratios (AORs) of the associations between sociodemographic measures and being present on the scene of and injured in a mass shooting. All analyses undertaken to generate population and relational inferences were weighted to achieve representativeness of US adults.

**Results:**

Of the 10 000 respondents included in the analysis, 51.34% (95% CI, 50.27%-52.40%) were female. In terms of race and ethnicity, 3.04% (95% CI, 2.71%-3.38%) were Asian, 12.46% (95% CI, 11.81%-13.12%) were Black, 16.04% (95% CI, 15.10%-16.98%) were Hispanic, 62.78% (95% CI, 61.73%-63.84%) were White, and 5.67% (95% CI, 5.23%-6.11%) were other race or ethnicity. The findings indicated that 6.95% (95% CI, 6.39%-7.50%) of respondents were present at the scene of a mass shooting, and 2.18% (95% CI, 1.85%-2.50%) sustained physical injuries, such as being shot or trampled, during such incidents. A total of 54.89% of mass shootings to which respondents were exposed occurred in 2015 or more recently, and 76.15% took place in respondents’ local communities. Mass shootings were most likely to occur in neighborhoods. Younger individuals (eg, AOR for Baby Boomer and Silent generations vs Generation Z, 0.12; 95% CI, 0.09-0.18) and males (AOR, 1.55; 95% CI, 1.29-1.85) were more likely to report exposure compared with those from older generations or female individuals, respectively. Black respondents reported higher rates of being present at mass shootings (AOR, 1.87; 95% CI, 1.49-2.34), while Asian respondents reported lower rates (AOR, 0.36; 95% CI, 0.19-0.66), compared with White respondents, but there were no racial and ethnic differences in injuries sustained.

**Conclusions and Relevance:**

The findings from this survey study of US adults underscore the extensive and often overlooked regular exposure to mass shootings in this country. The demographic disparities in exposure highlight the need for targeted interventions and support for the most affected groups, particularly younger generations and males. Understanding these patterns is essential for addressing the broader impacts of gun violence on public health and community well-being.

## Key Points

**Question:**

How prevalent is direct exposure to mass shootings in the US, and is risk equal across sociodemographic groups?

**Findings:**

In a survey study of 10 000 US adults, 7% reported having been present on the scene where 4 or more people were shot, which was more common among younger generations, males, and Black respondents. Two percent reported having been injured in a mass shooting—by being shot, trampled, or experiencing related injuries—which was more common among younger generations and males.

**Meaning:**

These findings underscore the extensive and often overlooked regular exposure to mass shootings in US society, which calls for targeted interventions designed to reduce violence.

## Teaser

This survey study estimates the prevalence of direct exposure to mass shootings among US adults and identifies the sociodemographic groups most exposed.

## Introduction

Mass shootings—incidents where 4 or more individuals are shot with a firearm—have emerged as a unique category of violence in the US, marked by their frequency, severity, and widespread media coverage.^1^ Since 2014, there have been nearly 5000 mass shootings documented nationwide, with more than 500 occurring annually since 2020, according to the Gun Violence Archive.^2^ The scale of these events has prompted concerns about the extent to which US residents are exposed to this form of violence. However, defining a mass shooting is not straightforward. Some definitions exclude nonfatal incidents or shootings associated with criminal activities such as gang conflicts or felony assaults, focusing instead on events involving indiscriminate shooters in public spaces.^3^ Others include these cases to explore how mass shootings intersect with other forms of gun violence, particularly in racial and ethnic minority communities affected by structural disadvantages and systemic racism.^4^ This lack of definitional clarity has contributed to inconsistent estimates of the prevalence of mass shootings and obscured understanding of the populations most affected.

Existing research has focused on the geographic distribution of mass shootings,^5^ psychological and physical impacts on survivors and their families,^6,7,8^ media exposure, and broader societal consequences.^9^ However, the literature has failed to capture how many US residents have been directly exposed to these incidents, either by being present at the scene or by sustaining physical injuries. Studies to date have often relied on small samples, collapsed mass shootings with other types of gun violence, or conflated indirect exposure—such as through media or personal connections—with direct exposure, which limits understanding of the phenomenon.^10,11,12,13,14^ Recent surveys, such as the 2023 Kaiser Family Foundation (KFF) survey^15^ and the 2023 *Economist*/YouGov survey,^16^ have suggested that gun violence is a common experience among US adults, but their broad definitions of exposure make it difficult to isolate mass shootings specifically. For example, the KFF survey found that 54% of respondents reported some form of personal or familial experience with gun violence, but this included threats with a gun, witnessing shootings, and being injured in non–mass shooting incidents.^15^ Similarly, the *Economist* and YouGov survey reported that 13% of respondents had been “personally affected by a mass shooting,” but the survey did not provide a clear definition of what constituted “personally affected” or “mass shooting.”^16^ As a result, existing data do not provide a precise estimate of direct exposure to mass shootings among the general population or the sociodemographic groups most at risk.

Based on a survey of 10 000 US adults (18 years or older) we estimate the prevalence and correlates of direct exposure to mass shootings in the US. We defined direct exposure as being present at the scene of a mass shooting or sustaining a physical injury from the event, whether by being shot, trampled, or experiencing another injury during the incident. Our survey captured detailed sociodemographic information, including age, gender, race and ethnicity, and socioeconomic status, to determine whether exposure varies across different groups.

## Methods

### Data

In January 2024, we contracted with an online market research firm (YouGov) to field a representative survey of adults in the US. Estimates from the Gun Violence Archive and National Crime Victimization Survey led us to generate a sample of 10 000 individuals residing in the US. Survey questions were designed based on prior research on gun violence exposure, namely the KFF survey of gun violence exposure.^15^ This study followed the American Association for Public Opinion Research (AAPOR) reporting guideline. The Institutional Review Board at Hamline University approved the study protocol. All survey respondents provided written informed consent.

The sample was derived from multistage matched design sampling. The target population was enumerated using a synthetic sampling frame—by age, educational attainment, gender, and race and ethnicity—based on several national sources of population data, including the US Census American Community Survey public use microdata file. Sample matching was undertaken by first generating a target sample drawn at random from the target population. A matched sample was then created based on selecting individuals to be interviewed matched to the target sample from a large pool of online opt-in panelists. Interviewed respondents were assigned weights using propensity scores, derived from a logistic regression model with age, educational attainment, gender, race and ethnicity, region, and 2020 presidential vote, to estimate inclusion in the sampling frame. Poststratified weights were then generated and used in all analyses to produce values designed to be representative of US adults.

Individuals who opt in to the online panel were sent a generic email with an invitation to participate in an online survey. They were prompted to click a link that leads them to a landing page that described the study, “Exposure to Gun Violence in the United States.” Upon providing written informed consent to take part in the study, respondents completed an online self-administered custom survey designed to yield information on exposure to gun violence. The survey contained multiple sections (eg, demographic, economic, firearm exposure, political, and social) and was completed in approximately 12 minutes by respondents. The number of questions varied by participants based on the degree of their various exposure. Participants were compensated by the market research firm for their survey completion using a point system. The eMethods in Supplement 1 reports additional information on data quality, sampling, generalizability checks, and assessment of nonrandom sample selection.

### Measures of Direct Exposure to Mass Shootings

This study was concerned with direct exposure to mass shootings, which were defined as “gun-related crimes where 4 or more people are shot in a public space, such as a school, shopping mall, workplace, or place of worship.” This definition was a compromise between the Congressional Research Service’s definition of a mass public shooting^17^ and the Gun Violence Archive’s mass shooting definition,^2^ designed to be inclusive of individuals who were injured and accessible to the public.

To capture direct exposure, respondents were asked: “Have you personally ever been physically present on the scene of a mass shooting in your lifetime?” The survey clarified physically present as “in the immediate vicinity of where the shooting occurred at the time it occurred, such that bullets were fired in your direction, you could see the shooter, or you could hear the gunfire.” The item included yes and no response categories, coded 1 and 0, respectively, along with an option of “don’t know.” Respondents who did not answer yes were considered as having no direct exposure to mass shootings and transitioned to a different section of the survey.

Respondents who answered yes to being physically present on the scene of a mass shooting were also asked additional questions about the incident. To measure injury exposure, respondents were asked, “Were you physically injured in the incident? (which could include being shot, trampled, or something else that caused physical injury).” The item included yes and no response categories, coded 1 and 0, respectively. Questions concerning the mental health consequences of exposure were also asked in the survey; this study focused on physical injuries rather than perceived short- and long-term psychological impacts.

Year of occurrence captures the year in which the incident occurred. Three-fourths of respondents were able to provide the year; no imputation was conducted for missing responses. The question concerning local community asked whether the shooting occurred in their local community, which was defined as “a geographic area in which you reside or to which you feel especially close, such as a neighborhood, small city or area in a larger city, or place where you spend a large amount of your time, such as a workplace, place of worship, or recreational area.” Location of occurrence measured places where mass shootings commonly occur, such as neighborhoods, schools, outdoor events, or workplaces (see the eMethods in Supplement 1 for the definitions of locations). Media coverage taps whether the respondent believed the incident was “covered widely by news media,” which we defined as “by national news media or by media beyond your city.”

### Measures of Correlates

Well-known indicators of generational cohorts differentiated by birth years include the Silent generation (before 1946), Baby Boomer generation (1946-1964), Generation X (1965-1980), Millennial generation (1981-1996), and Generation Z (1997 or more recently). The Silent and Baby Boomer generations were pooled owing to sparsity in the direct exposure outcomes.

Gender refers to self-identification as male or female. Respondents could self-identify with several racial or ethnic groups, which included Asian, Black, Hispanic, White, or other, the latter of which included American Indian or Alaska Native, Middle Eastern, Native Hawaiian or Other Pacific Islander, and multiple races and/or ethnicities. White respondents constituted the reference group.

Socioeconomic correlates included educational attainment and income. Educational attainment contained 4 response categories, including high school diploma or lower, some college, 4-year college degree, and graduate degree. These items were dummy coded, and the lowest educational attainment category was used as the reference group. Income captured 16 categories of annual family income, pooled into quintiles that included less than $20 000, $20 000 to $39 999, $40 000 to $69 999, $70 000 to $119 999, and $120 000 or greater. These items were dummy coded, and the lowest family income quintile was used as the reference group. Seven percent of respondents declined to report income; they were coded 0 for income and assigned a value of 1 in a dummy variable for nonresponse, where valid responses were coded 0. Control variables included geographic region of residence and news interest; both are described in the eMethods in Supplement 1.

### Statistical Analysis

The purpose of this study was to determine the prevalence of direct exposure to mass shootings and the associated features of the incidents, including the characteristics of the population more or less likely to encounter such violence. Univariable statistics were reported for the outcome variables to estimate the prevalence of US adults with direct exposure to mass shootings. Multivariable logistic regression was used to generate adjusted odds ratios (AORs) of the associations between sociodemographic measures and being present on the scene of and injured in a mass shooting. Two-tailed significance tests are reported and used to generate 95% CIs. All analyses undertaken to generate population and relational inferences were weighted to achieve representativeness of US adults and conducted using Stata, version 18.0 (StataCorp LLC); all analytic output can be found in the eAppendix in Supplement 1.

## Results

Table 1 provides descriptive statistics of the analytic sample of 10 000 US adults. Respondents closely approximated the characteristics of adults from the American Community Survey (eMethods in Supplement 1). The sample included 3.88% (95% CI, 3.49%-4.27%) from the Silent generation, 28.13% (95% CI, 27.18%-29.08%) from the Baby Boomer generation, 25.34% (95% CI, 24.42%-26.26%) from Generation X, 27.89% (95% CI, 26.94%-28.84%) from the Millennial generation, and 14.76% (95% CI, 13.97%-15.55%) from Generation Z. Female respondents were slightly more likely (51.34%; 95% CI, 50.27%-52.40%) to be represented compared with male respondents (48.66%; 95% CI, 47.60%-49.73%). Self-reported race and ethnicity included 3.04% (95% CI, 2.71%-3.38%) Asian, 12.46% (95% CI, 11.81%-13.12%) Black, 16.04% (95% CI, 15.10%-16.98%) Hispanic, 62.78% (95% CI, 61.73%-63.84%) White, and 5.67% (95% CI, 5.23%-6.11%) other. High school diploma or lower was the highest level of education for 38.39% (95% CI, 37.33%-39.45%) of respondents; 27.86% (95% CI, 26.91%-28.80%) completed some college but short of a baccalaureate degree; 21.46% (95% CI, 20.64%-22.28%) completed a 4-year degree; and 12.30% (95% CI, 11.65%-12.94%) completed a graduate degree. The annual family income for 23.13% (95% CI, 11.65%-12.94%) of the sample was below $20 000; 19.45% (95% CI, 18.60%-20.30%) of the sample reported $20 000 to $39 999; 21.18% (95% CI, 20.32%-22.05%) reported $40 000 to $69 999; 21.41% (95% CI, 20.55%-22.27%) reported $70 000 to $119 999; and 14.82% (95% CI, 14.09%-15.55%) reported $120 000 or more.

**Table 1.** Characteristics of Sample

Characteristic	Respondents, % (95% CI)^a^
Generation	
Silent	3.88 (3.49-4.27)
Boomer	28.13 (27.18-29.08)
X	25.34 (24.42-26.26)
Millennial	27.89 (26.94-28.84)
Z	14.76 (13.97-15.55)
Gender	
Female	51.34 (50.27-52.40)
Male	48.66 (47.60-49.73)
Race and ethnicity	
Asian	3.04 (2.71-3.38)
Black	12.46 (11.81-13.12)
Hispanic	16.04 (15.10-16.98)
White	62.78 (61.73-63.84)
Other^b^	5.67 (5.23-6.11)
Educational attainment	
High school diploma or lower	38.39 (37.33-39.45)
Some college	27.86 (26.91-28.80)
4-y Degree	21.46 (20.64-22.28)
Graduate degree	12.30 (11.65-12.94)
Income, US $	
≤19 999	23.13 (11.65-12.94)
20 000-39 999	19.45 (18.60-20.30)
40 000-69 999	21.18 (20.32-22.05)
70 000-119 999	21.41 (20.55-22.27)
≥120 000	14.82 (14.09-15.55)

^a^ Includes 10 000 respondents. All values are weighted to achieve representatives for US adults.

^b^ Includes respondents who self-identified as American Indian or Alaska Native, Middle Eastern, Native Hawaiian or Other Pacific Islander, and multiple races and/or ethnicities.

Table 2 reveals the prevalence of direct exposure to mass shootings in the US. A total of 6.95% (95% CI, 6.39%-7.50%) of respondents indicated they had been present on the scene of a mass shooting in their lifetime. Physical injuries that occurred during a mass shooting, which could include being shot, being trampled, or other causes of injury, were reported by 2.18% (95% CI, 1.85%-2.50%) of respondents.

**Table 2.** Prevalence of Direct Exposure to and Associated Features of Mass Shootings

Feature	Respondents, %^a^
**Full sample (N = 10 000)**	
Present on scene (95% CI)	6.95 (6.39-7.50)
Injured in incident (95% CI)	2.18 (1.85-2.50)
**Exposure subsample (n = 696)**	
Present on scene	100
Injured in incident	31.32
Year of occurrence, mean (SD)	2010.85 (13.05)
Local community	76.15
Location of occurrence	
Neighborhood	34.69
Bar or restaurant	12.38
K-12 school	12.09
Shopping outlet	11.51
Concert or outdoor event	11.05
College or university	6.98
Office or workplace	3.06
Place of worship	2.59
Movie theater	2.23
Park or public transit	2.21
Other	1.20
Covered by media	44.68

^a^ Based on respondent self-reports. All values are weighted to achieve representativeness of US adults.

The subsample of respondents with direct exposure to a mass shooting also shared the associated features of the incident. About two-third of respondents were present on the scene but not injured. While the mean year of the mass shooting occurrence was 2011 (SD, 13.05 years), more than half of the respondents indicated exposure in the last 10 years (54.89% in 2015 or more recently); the earliest recorded mass shooting took place in 1960 while the most recent was December 2023.

Most mass shootings (76.15%) occurred in what the respondent considered their local community. The modal location of occurrence—reported by 34.69% of the exposed subsample—was identified by respondents as a neighborhood. Bars or restaurants (12.38%), schools (12.09%), shopping outlets (11.51%), and concerts or outdoor events (11.05%) were the next most common. These 5 locations accounted for more than 80% of direct exposure to mass shootings. Descriptive statistics by location of occurrence are found in eTable 1 in Supplement 1. In the exposed subsample, 44.68% of respondents reported that the mass shooting to which they were directly exposed was covered widely by the news media. eTable 2 in Supplement 1 contains descriptive statistics for shootings perceived to receive media coverage or not.

Table 3 demonstrates that risk of exposure to mass shootings was unequal across sociodemographic groups. A generational gradient was observed for each type of exposure. Respondents born in earlier generations—especially the Baby Boomer and Silent generations (born 1964 or earlier)—were less likely to report any experience with mass shootings than respondents born in recent generations, despite greater time periods of risk for exposure (AOR for Baby Boomer and Silent generations vs Generation Z, 0.12; 95% CI, 0.09-0.18).

**Table 3.** Multivariable Logistic Regression and AORs Estimating Personal Exposure to Mass Shootings Based on Sociodemographic Variables^a^

Covariate	AOR (95% CI)	
	Present on scene	Injured in incident
Generation		
Z	1 [Reference]	1 [Reference]
Millennial	0.77 (0.61-0.96)	0.67 (0.47-0.97)
X	0.32 (0.24-0.42)	0.17 (0.10-0.29)
Baby Boomer and Silent	0.12 (0.09-0.18)	0.02 (0.004-0.05)
Gender		
Female	1 [Reference]	1 [Reference]
Male	1.55 (1.29-1.85)	1.88 (1.35-2.62)
Race and ethnicity		
Asian	0.36 (0.19-0.66)	0.43 (0.17-1.07)
Black	1.87 (1.49-2.34)	1.22 (0.82-1.82)
Hispanic	1.02 (0.77-1.35)	0.79 (0.49-1.27)
White	1 [Reference]	1 [Reference]
Other	1.02 (0.71-1.44)	0.56 (0.28-1.11)
Educational attainment		
High school diploma or lower	1 [Reference]	1 [Reference]
Some college	0.89 (0.71-1.11)	0.67 (0.43-1.04)
4-y Degree	1.12 (0.88-1.43)	1.03 (0.66-1.59)
Graduate degree	1.28 (0.96-1.71)	1.19 (0.71-1.99)
Income, US $		
<20 000	1 [Reference]	1 [Reference]
20 000-39 999	0.94 (0.68-1.29)	0.84 (0.47-1.50)
40 000-69 999	0.82 (0.61-1.12)	0.90 (0.53-1.53)
70 000-119 999	0.89 (0.65-1.20)	0.98 (0.59-1.65)
≥120 000	1.03 (0.75-1.43)	1.00 (0.58-1.72)

Abbreviation: AOR, covariate-adjusted odds ratio.

^a^ Includes 10 000 respondents. All values are weighted to achieve representatives for US adults. Confidence intervals are derived from robust standard errors. Model adjusts for media consumption, region and/or state, and missing responses to income.

Males maintained greater odds than females in reporting being present on the scene of a mass shooting (AOR, 1.55; 95% CI, 1.29-1.85) and being physically injured in a mass shooting (AOR, 1.88; 95% CI, 1.35-2.62). Race and ethnicity differences in exposure were mixed. White and Hispanic respondents were just as likely to report direct exposure to mass shootings (AOR for Hispanic vs White respondents, 1.02; 95% CI, 0.77-1.35). Black respondents were more likely (AOR, 1.87; 95% CI, 1.49-2.34) and Asian respondents were less likely (AOR, 0.36; 95% CI, 0.19-0.66) than White respondents to report being present on the scene of a mass shooting, but no racial or ethnic differences were observed for physical injuries.

There were no differences in direct exposure to mass shootings by socioeconomic status. Risk of exposure was also statistically indistinguishable across all 4 levels of educational attainment as well as the income quintiles. The estimated probabilities for the covariates (generation, gender, and race and ethnicity) associated with direct exposure to mass shooting, holding all covariates at their mean values, can be found in eTable 3 in Supplement 1.

## Discussion

Mass shootings are a public health concern in the US, distinguished by their high casualty rates, perceived randomness, and substantial impact on both survivors and the broader community.^3^ Although extensive research has documented the consequences of gun violence overall,^18^ less attention has been devoted to understanding the prevalence and distribution of direct exposure to mass shootings. This survey study addressed this gap by estimating the prevalence of direct exposure to mass shootings and examining sociodemographic differences in exposure rates, providing new insights into who is most affected by them.

Exposure to mass shootings is more pervasive than previously understood.^15^ Approximately 1 in 15 US residents reported being physically present during a mass shooting, while 2.18% reported sustaining physical injuries during such events. These results suggest that mass shootings are not isolated tragedies but rather a reality that reaches a substantial portion of the population in their lifetime.

Younger individuals, particularly the Millennial generation and Generation Z, were more likely to report being physically present on the scene and/or injured in a mass shooting compared with older generations. This generational difference could be partly attributable to the increasing frequency of mass shootings over time. These results should not be interpreted as evidence that a particular generation was disproportionately targeted by mass shootings at the expense of another. Rather, they highlight the need for future research to examine how generational differences in exposure intersect with trends in the frequency and lethality of mass shootings.^3^

Males were more likely than females to report direct exposure to mass shootings, which is consistent with broader patterns of gun violence exposure, where males, particularly young males, face higher risks.^19^ Black respondents reported higher rates of being present at mass shootings compared with White respondents, consistent with longstanding research on the racial disparities in exposure to gun violence.^20^ However, our data did not show that these racial differences extended to the likelihood of sustaining injuries. This finding could reflect broader exposure to neighborhood violence in Black than White communities; eTable 1 in Supplement 1 offers suggestive evidence in support of such differences.

Our findings highlight the substantial reach of mass shootings in US society. This widespread exposure underscores the need for comprehensive public health strategies to address the broad and enduring impacts of mass shooting exposure. Being present at a mass shooting can have profound physical and psychological consequences, not only for survivors who sustain injuries but also for those who witness the violence or experience the aftermath.^12,13^ These incidents disrupt community cohesion and generate long-lasting trauma that reverberates through families, neighborhoods, and workplaces.

The lack of significant variation in exposure across socioeconomic groups points to the fact that mass shootings are not confined to traditionally high-risk populations. Instead, they occur in diverse social and geographic contexts,^5^ affecting a wide array of individuals and communities. Public health responses must be adaptable and inclusive, capable of addressing the needs of a broad spectrum of the population. Prevention efforts should focus on reducing the incidence of mass shootings overall,^3^ while postincident support must prioritize building sustainable, community-wide systems of care that can respond effectively when these tragedies occur. Any such responses should not come at the expense of efforts to reduce and cope with everyday gun violence that does not involve 4 or more individuals who were injured.

### Limitations

Findings on the prevalence of direct exposure and associated correlates are subject to well-known limitations of survey research. Although survey prompts defined mass shootings and direct exposure, recall bias and cohort-specific experiences related to growing up in an era characterized by heightened awareness of gun violence could contribute to the generational gradient observed. Invariance in injury may mask differential forms of exposure by location and type of mass shooting, as well as the circumstances under which these incidents occur, including measurement of lifetime exposure (eg, shorter vs longer recall periods) and types of physical injuries (eg, shot vs trampled).

## Conclusions

This survey study offers evidence of wide-reaching direct exposure and experience with mass shootings in the US. Ongoing research and data collection are essential to understanding the full scope of mass shootings and their impact on US society. Future research should further investigate how direct exposure to these incidents shapes individuals’ health and well-being, with particular attention to the broader community and the potential for systemic interventions that promote safety and resilience.

## Notes

Supplement 1. **eMethods.** Supplemental Data and Analysis **eTable 1.** Descriptive Statistics by Location of Mass Shooting Occurrence **eTable 2.** Descriptive Statistics by Whether a Mass Shooting Attracted Wide Media Coverage **eTable 3.** Estimated Probabilities of Direct Exposure to Mass Shootings by Generation, Gender, and Race and Ethnicity **eAppendix.** Stata Log File

Supplement 2.
Data Sharing Statement
