# Supplementary material for: Direct Exposure to Mass Shootings Among US Adults
Source: JAMA Netw Open. 2025 Mar 7;8(3):e250283. doi: 10.1001/jamanetworkopen.2025.0283 (PMC11889466; doi:10.1001/jamanetworkopen.2025.0283)
Supplement: Supplement 1. — eMethods. Supplemental Data and Analysis eTable 1. Descriptive Statistics by Location of Mass Shooting Occurrence eTable 2. Descriptive Statistics by Whether a Mass Shooting Attracted Wide Media Coverage eTable 3. Estimated Probabilities of Direct Exposure to Mass Shootings by Generation, Gender, and Race and Ethnicity eAppendix. Stata Log File [file jamanetwopen-e250283-s001.pdf]

Supplementary Online Content

Pyrooz DC, Densley JA, Peterson JK. Direct exposure to mass shootings among US adults. *JAMA Netw Open.* 2025;8(3):e250283.
doi:10.1001/jamanetworkopen.2025.0283

eMethods. Supplemental Data and Analysis
eTable 1. Descriptive Statistics by Location of Mass Shooting Occurrence
eTable 2. Descriptive Statistics by Whether a Mass Shooting Attracted Wide Media Coverage
eTable 3. Estimated Probabilities of Direct Exposure to Mass Shootings by Generation, Gender, and Race and Ethnicity
eAppendix. Stata Log File

This supplementary material has been provided by the authors to give readers additional information about their work.

© 2025 Pyrooz DC et al. *JAMA Network Open*.

eMethods. Supplemental Data and Analysis
Supplemental Information on Location of Exposure

Location of exposure included a range of places where mass shootings commonly occur, which were worded as follows: (1) neighborhood (home, backyard, apartment), (2) school, (3) college/university, (4) place of worship, (5) shopping mall, grocery store, commercial, (6) bar, restaurant, nightclub, (7) move theater, (8) outdoor concert or event, (9) office, workplace, or government building, and (10) other. Based on "other" responses, we categorized reports of shootings that occurred at hotels in the "shopping outlets" (5) and downtown or parades as "outdoor events" (8). Enough responses listed parks, highways, and public transit to warrant a standalone category (11). Responses that listed shootings in war zones were removed given the focus on domestic mass shootings.

Measures of Control Variables

Additional variables were included as controls. Geographic variables accounted for differences in exposure from larger states and smaller regions. Respondents residing in states with populations exceeding 15 million contained large enough representation in the survey to warrant differentiation. These states include *California*, *Florida*, *New York*, and *Texas*, where respondents in the remaining states and District of Columbia were grouped into U.S. Census defined regions of *West*, *Midwest*, *South*, and *Northeast*.

News interest, which captures the frequency with which respondents observe the news, including *hardly at all / don't know* (15.0%), *only now and then* (15.5%), *some of the time* (30.2%), and *most of the time* (39.4%), was used as a control variable. These items were dummy coded and the least news interest category was used as the reference group.

Sampling, Quality Checks, Generalizability, and Non-Random Selection

Online, opt-in panels offer many advantages to researchers, including flexibility, timeliness, and cost-effectiveness. They are not immune from concerns about generating population and relational inferences. A large N sample, combined with multi-stage, matched design sampling, minimize some of these concerns. Still, the goal is to model the population of interest (i.e., US adults) with non-probability sampling, which it why it remains important to consider sampling procedure, quality check, generalizability, and non-random selection in a custom survey that aims to generate information on direct exposure to mass shootings.

The sample of 10,000 was generated through multi-stage, matched design sampling. YouGov maintains about six million panelists in the United States, several hundred thousand of whom are very active. It is from this panel that individuals are selected to be invited to complete a survey, which is determined via project specifications and algorithmically relative to ongoing omnibus and custom surveys being fielded. Of the 13,425 panelists who clicked the link, some were removed because they only partially

© 2025 Pyrooz DC et al. *JAMA Network Open*.

completed the survey, were screened out due to a lack of qualification, were over target quotas for a given socio-demographic group, or due to quality checks.

Quality checks were undertaken by YouGov to screen out respondents who did not meet quality control standards (e.g., speed throughs, attention checks, non-sensical responses, and duplicate internet protocol addresses). Additional quality checks were undertaken by the authors in examining responses to supplemental questions about local and personal exposure to mass shootings. Inquiries into the place in which a personal exposure incident occurred and reason for presence at the location resulted in removing a small number of incidents in which respondents were not present on the scene (*n*=4), cases in which incidents occurred in war zones as part of military service (*n*=16), or cases that occurred outside of the United States that appeared independent of military service (*n*=20). There were also six instances where local community exposure occurred outside of the United States.

The descriptive characteristics of the sample closely approximate national estimates of adults derived from the 2022 American Community Survey 1-year estimates. The sample is 3.9% from the Silent Generation (ACS = 6.6%), 28.1% from the Baby Boomer Generation (ACS = 25.4%), 25.3% from Generation X (ACS = 25.1%), 27.9% from Millennial Generation (ACS = 27.5%), and 14.8% from Generation Z (ACS = 15.4%). Females are slightly more likely (51.3%) to be represented as respondents than males (48.7%), though consistent with ACS estimates (females = 50.9%, males = 49.1%). The predominant racial/ethnic groups in the United States were consistently represented in the sample in relation to ACS estimates: 62.8% White (ACS = 63.3%), 16.0% Hispanic (ACS = 17.2%), and 12.5% Black (ACS = 11.9%). Levels of educational attainment and regional representation in the study sample also closely match ACS data. High school diploma or lower was the highest level of education for 39.4% of respondents (ACS = 37.7%), 27.9% completed some college but fell short of a baccalaureate degree (ACS = 29.3%), 21.5% completed a 4-year degree (ACS = 20.6%), and 12.3% completed graduate degree (ACS = 12.4%). Income categories could not be directly compared owing to how YouGov categorized family income. California, Florida, New York, and Texas respondents composed 11.0%, 7.1%, 7.8%, and 9.2%, respectively, which closely proxies the ACS estimates of 11.7%, 6.9%, 6.0%, and 8.7%, respectively.

Whereas YouGov's matched sample design induces similarity in demographic, socioeconomic, and regional characteristics, alternative indicators may produce different results from probability samples derived from random digit dialing and housing units. Therefore, it is important to assess the generalizability of the inferences reached in this study by comparing findings to established probability samples. Since this is the first instance of surveying a national sample on exposure to mass shootings, it was necessary to use an alternative indicator as a substantively relevant generalizability check. Fear of victimization is regularly asked as part of Gallup polling and the General Social Survey. An identical item was administered to respondents in this YouGov sample, which read as follows: "Is there any area near where you live – that is, within a mile – where you would be afraid to walk alone at night?" The results were consistent across samples. In this YouGov sample, 39.8% of respondents said "yes." Gallup

© 2025 Pyrooz DC et al. *JAMA Network Open*.

administered this question in October of 2021 and 2023, where 37% and 40% of respondents answered in the affirmative (https://news.gallup.com/poll/1603/crime.aspx). The General Social Survey administered this question in 2022, where 37% of respondents stated “yes” (https://gssdataexplorer.norc.org/trends?category=Civil%20Liberties&measure=fear). Overall, these findings suggest YouGov’s matched sample design is capable of yielding generalizable inferences.

To address the prospect of non-random selection into a custom survey, which was titled “Exposure to Gun Violence in the United States,” we determined if similar results could be obtained using an omnibus survey. An omnibus survey is similar to a custom survey in that it is generated using the same sampling strategy but differs in that a wide range of questions are included, rendering respondents unaware of the content upon agreeing to complete a survey. We inserted the personal exposure to a mass shooting question into an omnibus survey fielded the same month as the custom survey to 3,000 respondents, finding that 7.77 percent of respondents indicated that they were present on the scene of a mass shooting. This estimate closely approximates the observation from the custom survey, which in combination with being derived from a separate sample, enhances confidence in the findings.

Descriptive Statistics by Location of Direct Exposure to Mass Shootings

eTable 1 provides a breakdown of the socio-demographic characteristics of respondents subject to direct exposure to mass shootings by location of its occurrence. It demonstrates the generational gradient and gender differences across location. Race/ethnic differences vary depending on location (e.g., neighborhood v. shopping outlet). Socioeconomic differences also vary more when location is disaggregated than when it is not.

Descriptive Statistics by Mass Shootings that Attracted Wide Media Coverage

eTable 2 provides descriptive statistics partitioned by respondents who indicated the mass shooting to which they were directly exposed received wide media coverage. The differences, as a whole, were not stark, but tended to align with research suggesting that mass media declines to coverage the mass shootings encountered by more marginalized populations. For instance, shootings that did not receive coverage were experienced by respondents who were more likely to be Black or Hispanic than White, maintained lower educational attainment and family income, and occurred in neighborhoods rather than commercial or government places.

Predicted Probabilities of Direct Exposure to Mass Shootings by Generation, Gender, and Race/Ethnicity

The predicted probabilities (and 95% confidence intervals) of direct exposure and injury exposure to mass shootings are reported in eTable 3 for the three factors associated with the outcomes.

© 2025 Pyrooz DC et al. *JAMA Network Open*.

eTable 1. Descriptive Statistics by Location of Mass Shooting Occurrence

	Full Sample n=10,000 %	Neighbor-hood n=247 %	Bar / Restaurant n=88 %	K-12 School n=81 %	Shopping Outlet n=85 %	Concert / Outdoor Event n=76 %	Other Location n=119 %
Generation							
Silent	3.9	0.3	0.0	0.8	0.0	0.0	0.0
Boomer	28.1	11.4	5.3	8.6	6.4	7.9	7.5
X	25.3	19.1	14.9	24.3	12.6	16.0	17.3
Millennial	27.9	43.9	54.0	34.7	52.4	42.4	44.6
Z	14.8	25.3	25.8	31.6	28.6	33.7	30.6
Gender							
Female	51.3	39.8	30.7	31.5	40.3	41.3	42.5
Male	48.7	60.2	69.3	68.5	59.7	58.7	57.5
Race/Ethnicity							
White	62.8	48.0	56.5	50.7	59.1	57.3	60.7
Black	12.5	22.6	22.0	23.3	19.4	14.9	13.3
Hispanic	16.0	20.1	18.0	22.4	16.0	20.9	15.9
Asian	3.0	2.0	0.0	0.0	1.9	1.9	3.0
Other	5.7	7.4	3.6	3.7	3.5	5.0	7.2
Education							
High or lower	38.4	44.5	39.4	44.4	21.9	30.2	24.7
Some college	27.9	20.7	22.6	25.7	33.0	35.3	29.3
4-year degree	21.5	21.9	27.9	19.5	33.1	20.3	28.5
Graduate degree	12.3	12.9	10.1	10.4	12.0	14.2	17.6
Income							
$19,999 or lower	23.1	32.2	9.7	26.9	13.3	22.9	19.0
$20,000 to $39,999	19.5	17.0	21.8	12.6	21.1	13.2	19.4
$40,000 to $69,999	21.2	19.2	18.8	25.0	16.5	21.0	12.7
$70,000 to $119,999	21.4	17.8	27.9	14.7	25.8	23.4	26.8
$120,000 or higher	14.8	13.8	21.8	20.9	23.3	19.6	22.2
Region							
States < 15m residents	64.8	54.6	46.7	51.0	52.9	37.6	55.9
California	11.0	20.2	18.6	17.1	10.6	36.5	12.6
Florida	7.1	6.0	6.5	3.4	3.9	2.9	6.3
New York	7.8	15.0	14.8	18.2	19.8	8.2	13.9
Texas	9.2	4.2	13.4	10.3	12.8	14.9	11.4
News Interest							
Hardly at all/don't know	15.0	16.1	4.1	8.7	13.0	13.9	8.3
Only now and then	15.5	17.0	19.8	17.2	10.6	14.3	9.1
Some of the time	30.2	32.2	42.5	43.9	30.4	37.5	43.5
Most of the time	39.4	34.7	33.6	30.2	46.0	34.3	39.1

© 2025 Pyrooz DC et al. *JAMA Network Open*.

eTable 2. Descriptive Statistics by Whether a Mass Shooting Attracted Wide Media Coverage

	Media Coverage 310 %	No Media 385 %
Generation		
Silent	0.5	0.0
Boomer	6.9	10.0
X	12.5	22.1
Millennial	50.3	40.7
Z	29.9	27.2
Gender		
Female	38.8	38.0
Male	61.2	61.2
Race/Ethnicity		
White	60.9	48.4
Black	17.3	21.6
Hispanic	16.5	21.0
Asian	2.0	1.3
Other	3.2	7.8
Education		
High or lower	30.1	40.9
Some college	24.4	27.6
4-year degree	28.7	21.4
Graduate degree	16.9	10.2
Income		
$19,999 or lower	19.0	26.6
$20,000 to $39,999	14.1	20.3
$40,000 to $69,999	18.6	18.5
$70,000 to $119,999	27.1	17.6
$120,000 or higher	21.3	17.0
Region		
States < 15m residents	19.0	26.6
California	14.1	20.3
Florida	18.6	18.5
New York	27.1	17.6
Texas	21.3	17.0
News Interest		
Hardly at all / Don't know	5.7	16.6
Only now and then	12.1	17.2
Some of the time	39.2	35.8
Most of the time	43.0	30.5
Injured in Incident	43.4	21.6
Year of Occurrence	2009.5	2012.5
Local Community	80.4	72.8
Neighborhood	25.3	42.3
Bar/Restaurant	16.5	9.09
K-12 School	13.3	11.1
Shopping Outlet	11.9	11.17
Concert/Outdoor Event	7.5	13.9

© 2025 Pyrooz DC et al. *JAMA Network Open*.

eTable 3. Estimated Probabilities of Direct Exposure to Mass Shootings by Generation, Gender, and Race and Ethnicity

	Present on the Scene of a Mass Shooting		Physically Injured in a Mass Shooting	
	Margin	[95% CI]	Margin	[95% CI]
Gen Z	0.136	[0.115, 0.157]	0.056	[0.041, 0.071]
Millennial	0.108	[0.096, 0.121]	0.039	[0.031, 0.046]
Gen X	0.049	[0.040, 0.059]	0.010	[0.006, 0.015]
Boomer/Silent	0.019	[0.014, 0.025]	0.001	[0.000, 0.002]
Female	0.056	[0.049, 0.063]	0.015	[0.011, 0.019]
Male	0.082	[0.074, 0.090]	0.027	[0.022, 0.032]
White	0.065	[0.058, 0.072]	0.023	[0.019, 0.028]
Black	0.111	[0.094, 0.128]	0.028	[0.019, 0.037]
Hispanic	0.066	[0.052, 0.081]	0.019	[0.011, 0.026]
Asian	0.025	[0.011, 0.039]	0.010	[0.001, 0.019]
Other	0.066	[0.471, 0.085]	0.013	[0.005, 0.022]

Note: Derived from models reported in Table 2 (main text), holding all variables at their mean values.

© 2025 Pyrooz DC et al. *JAMA Network Open*.

eAppendix. Stata Log File

```
--------------------------------------------------------------------------------------
--------------------------
      name:  <unnamed>
       log:  /XXXXX/YouGov Survey/Exposure to MV/Analysis/log.smcl
  log type:  smcl
 opened on:  25 Jan 2025, 12:49:10

.
. *** Project: YouGov customized MV survey
. *** Author: David Pyrooz
. *** Date:       March 4, 2024 (start)
. ***             July 26, 2024 (revise)
. ***             November 18, 2024 (final)
. ***             January 25, 2025 (copy editor update)
. *** Sample: N=10,000 national + N=1,000 Minnesota (dropped)
.
. keep /* survey */       caseid weight ///
>          /* DVs */              mass_8 pres_injure ///
>          /* IVs */              age birthyr generation male white black hisp asian
native multi other other2
> ///
>                                         state educyrs income income_miss oneparent
pareduc pareduc_miss newsi
> nt ///
>                                         pres_when_year pres_local pres_place
pres_media

.
. set more off

.
. drop if weight==.
(1,000 observations deleted)

.
. svyset [pweight=weight]

Sampling weights: weight
             VCE: linearized
     Single unit: missing
        Strata 1: <one>
 Sampling unit 1: <observations>
           FPC 1: <zero>

.
.
. gen generation2 = generation

.         replace generation2 = 3 if generation==4                       /* pool
Boomer/Silent for sparsity */
(410 real changes made)

.
. gen race_cat = 0 if white==1
(3,724 missing values generated)
```

© 2025 Pyrooz DC et al. *JAMA Network Open*.

```
.         replace race_cat=1 if black==1
(1,422 real changes made)

.         replace race_cat=2 if hisp==1
(1,320 real changes made)

.         replace race_cat=3 if asian==1
(329 real changes made)

.         replace race_cat=4 if other2==1                                         /*
AI/PI, ME, mixed, other */
(653 real changes made)

.
. gen ca = 0

.         replace ca=1 if state==6
(1,001 real changes made)

. gen fl = 0

.         replace fl=1 if state==12
(745 real changes made)

. gen ny = 0

.         replace ny=1 if state==36
(816 real changes made)

. gen tx = 0

.         replace tx=1 if state==48
(922 real changes made)

. gen state_cat = 0

.         replace state_cat=1 if ca==1
(1,001 real changes made)

.         replace state_cat=2 if fl==1
(745 real changes made)

.         replace state_cat=3 if ny==1
(816 real changes made)

.         replace state_cat=4 if tx==1
(922 real changes made)

.
. gen region = .
(10,000 missing values generated)

.         /*west*/        replace region = 0 if
inlist(state,2,4,6,8,15,16,30,32,35,41,49,53,56)
(2,142 real changes made)
```

© 2025 Pyrooz DC et al. *JAMA Network Open*.

```
.         /*south*/       replace region = 1 if
inlist(state,1,5,10,11,12,13,21,22,24,28,37,45,47,48,51,54)
(3,893 real changes made)

.         /*midwest*/     replace region = 2 if
inlist(state,17,18,19,20,26,27,29,31,38,39,40,46,55)
(2,104 real changes made)

.         /*noreast*/     replace region = 3 if
inlist(state,9,23,25,33,34,36,42,44,50)
(1,861 real changes made)

.
. label define region 0 "west" 1 "south" 2 "midwest" 3 "northeast" 4 "ca" 5 "fl" 6
"ny" 7 "tx"

. gen state_region = .
(10,000 missing values generated)

.         replace state_region = 0 if region==0
(2,142 real changes made)

.         replace state_region = 1 if region==1
(3,893 real changes made)

.         replace state_region = 2 if region==2
(2,104 real changes made)

.         replace state_region = 3 if region==3
(1,861 real changes made)

.         replace state_region = 4 if ca==1
(1,001 real changes made)

.         replace state_region = 5 if fl==1
(745 real changes made)

.         replace state_region = 6 if ny==1
(816 real changes made)

.         replace state_region = 7 if tx==1
(922 real changes made)

. label val state_region "region"

.
. label define edu 0 "hs or lower" 1 "some college" 2 "4-year " 3 "post-grad"

.         recode educ (1/2=0) (3/4=1) (5=2) (6=3)
(0 changes made to educyrs)

. label val educ "edu"

.
. label define inc 0 "lowest quintile" 1 "2nd quintile" 2 "3rd quintile" 3 "4th
quintile" 4 "5th quintile"
```

© 2025 Pyrooz DC et al. *JAMA Network Open*.

```
.         recode income (.=0) (5000/15000=0) (25000/35000=1) (45000/65000=2)
(75000/110000=3) (135000/500000=4)
>
(10,000 changes made to income)

. label val income "inc"

.
. replace pres_when_year=. if mass_8==0   /* n=32 cases provided year */
(32 real changes made, 32 to missing)

. replace pres_local=0 if mass_8==0               /* n=9 cases said "yes" */
(9 real changes made)

. replace pres_place=99 if mass_8==0              /* n=21 non-military cases */
(37 real changes made)

. replace pres_media=0 if mass_8==0               /* n=21 cases */
(21 real changes made)

.
. tab pres_place, gen(place_)
/* location context o
> f mass shooting */

   Present - Place of incident |      Freq.     Percent        Cum.
-------------------------------+-----------------------------------
                  Neighborhood |        247        2.47        2.47
                        School |         81        0.81        3.28
            College/university |         42        0.42        3.70
              Place of worship |         19        0.19        3.89
Shopping mall or grocery store |         85        0.85        4.74
             Bar or restaurant |         88        0.88        5.62
                 Movie theatre |         17        0.17        5.79
      Outdoor concert or event |         76        0.76        6.55
           Office or workplace |         22        0.22        6.77
                         Other |          5        0.05        6.82
                            11 |         14        0.14        6.96
                     not asked |      9,304       93.04      100.00
-------------------------------+-----------------------------------
                         Total |     10,000      100.00

.         rename place_1 place_nhood

.         rename place_2 place_school

.         rename place_3 place_college

.         rename place_4 place_worship

.         rename place_5 place_shopping

.         rename place_6 place_bar

.         rename place_7 place_movie

.         rename place_8 place_event
```
© 2025 Pyrooz DC et al. *JAMA Network Open*.

```
.         rename place_9 place_workplace

.         rename place_10 place_other

.         rename place_11 place_park

.
.
. ***** Table 1: Descriptive statistics of the sample
. tab generation [aweight=weight], gen(gen)

 generation |      Freq.     Percent        Cum.
------------+-----------------------------------
          0 | 1,476.1329       14.76       14.76
          1 | 2,789.2778       27.89       42.65
          2 | 2,533.7098       25.34       67.99
          3 | 2,812.7495       28.13       96.12
          4 | 388.130087        3.88      100.00
------------+-----------------------------------
      Total |     10,000      100.00

. tab male [aweight=weight], gen(sex)

       male |      Freq.     Percent        Cum.
------------+-----------------------------------
          0 |    5,133.7       51.34       51.34
          1 |    4,866.3       48.66      100.00
------------+-----------------------------------
      Total |     10,000      100.00

. tab race_cat [aweight=weight]

   race_cat |      Freq.     Percent        Cum.
------------+-----------------------------------
          0 |    6,278.4       62.78       62.78
          1 |    1,246.1       12.46       75.25
          2 |   1,603.88       16.04       91.28
          3 | 304.364622        3.04       94.33
          4 | 567.255378        5.67      100.00
------------+-----------------------------------
      Total |     10,000      100.00

. tab educ [aweight=weight], gen(ed)

     educyrs |      Freq.     Percent        Cum.
-------------+-----------------------------------
          10 | 586.870539        5.87        5.87
          12 | 3,252.1895       32.52       38.39
          13 |1,856.47721       18.56       56.96
          14 |  929.14279        9.29       66.25
          16 |    2,145.7       21.46       87.70
          18 |   1,229.62       12.30      100.00
-------------+-----------------------------------
       Total |     10,000      100.00

. tab income [aweight=weight], gen(inc)
```
© 2025 Pyrooz DC et al. *JAMA Network Open*.

```
         income |      Freq.     Percent        Cum.
----------------+-----------------------------------
lowest quintile | 2,313.2551       23.13       23.13
   2nd quintile | 1,944.8775       19.45       42.58
   3rd quintile |2,118.37435       21.18       63.77
   4th quintile | 2,141.3449       21.41       85.18
   5th quintile | 1,482.1481       14.82      100.00
----------------+-----------------------------------
          Total |     10,000      100.00

. tab state_cat [aweight=weight]

  state_cat |      Freq.     Percent        Cum.
------------+-----------------------------------
          0 | 6,481.1479       64.81       64.81
          1 | 1,103.7418       11.04       75.85
          2 | 713.935439        7.14       82.99
          3 | 781.210726        7.81       90.80
          4 | 919.964062        9.20      100.00
------------+-----------------------------------
      Total |     10,000      100.00

. tab newsint [aweight=weight]

      Political Interest |      Freq.     Percent        Cum.
-------------------------+-----------------------------------
Hardly at all/Don't know |  1,496.182       14.96       14.96
       Only now and then | 1,545.3216       15.45       30.42
        Some of the time | 3,016.1281       30.16       60.58
        Most of the time |3,942.36836       39.42      100.00
-------------------------+-----------------------------------
                   Total |     10,000      100.00

.
. foreach var of varlist gen1 gen2 gen3 gen4 gen5 sex1 sex2 asian black hisp white
other2 ed1 ed2 ed3 ed4 inc1
> inc2 inc3 inc4 inc5 {
  2.         svy: mean `var'
  3. }
(running mean on estimation sample)

Survey: Mean estimation

Number of strata =      1             Number of obs   = 10,000
Number of PSUs   = 10,000             Population size = 10,000
                                      Design df       =  9,999

--------------------------------------------------------------
             |             Linearized
             |       Mean   std. err.     [95% conf. interval]
-------------+------------------------------------------------
        gen1 |   .1476133   .0040146      .1397438    .1554828
--------------------------------------------------------------
(running mean on estimation sample)

Survey: Mean estimation
```
© 2025 Pyrooz DC et al. *JAMA Network Open*.

```
Number of strata =      1             Number of obs   = 10,000
Number of PSUs   = 10,000             Population size = 10,000
                                      Design df       =  9,999

--------------------------------------------------------------
             |             Linearized
             |       Mean   std. err.     [95% conf. interval]
-------------+------------------------------------------------
        gen2 |   .2789278   .0048449      .2694309    .2884247
--------------------------------------------------------------
(running mean on estimation sample)

Survey: Mean estimation

Number of strata =      1             Number of obs   = 10,000
Number of PSUs   = 10,000             Population size = 10,000
                                      Design df       =  9,999

--------------------------------------------------------------
             |             Linearized
             |       Mean   std. err.     [95% conf. interval]
-------------+------------------------------------------------
        gen3 |    .253371    .004698       .244162      .26258
--------------------------------------------------------------
(running mean on estimation sample)

Survey: Mean estimation

Number of strata =      1             Number of obs   = 10,000
Number of PSUs   = 10,000             Population size = 10,000
                                      Design df       =  9,999

--------------------------------------------------------------
             |             Linearized
             |       Mean   std. err.     [95% conf. interval]
-------------+------------------------------------------------
        gen4 |   .2812749   .0048418      .2717841    .2907658
--------------------------------------------------------------
(running mean on estimation sample)

Survey: Mean estimation

Number of strata =      1             Number of obs   = 10,000
Number of PSUs   = 10,000             Population size = 10,000
                                      Design df       =  9,999

--------------------------------------------------------------
             |             Linearized
             |       Mean   std. err.     [95% conf. interval]
-------------+------------------------------------------------
        gen5 |    .038813   .0019774       .034937    .0426891
--------------------------------------------------------------
(running mean on estimation sample)

Survey: Mean estimation
```

```
Number of strata =      1             Number of obs   = 10,000
Number of PSUs   = 10,000             Population size = 10,000
                                      Design df       =  9,999

--------------------------------------------------------------
             |             Linearized
             |       Mean   std. err.     [95% conf. interval]
-------------+------------------------------------------------
        sex1 |     .51337    .005431      .5027241    .5240159
--------------------------------------------------------------
(running mean on estimation sample)

Survey: Mean estimation

Number of strata =      1             Number of obs   = 10,000
Number of PSUs   = 10,000             Population size = 10,000
                                      Design df       =  9,999

--------------------------------------------------------------
             |             Linearized
             |       Mean   std. err.     [95% conf. interval]
-------------+------------------------------------------------
        sex2 |     .48663    .005431      .4759841    .4972759
--------------------------------------------------------------
(running mean on estimation sample)

Survey: Mean estimation

Number of strata =      1             Number of obs   = 10,000
Number of PSUs   = 10,000             Population size = 10,000
                                      Design df       =  9,999

--------------------------------------------------------------
             |             Linearized
             |       Mean   std. err.     [95% conf. interval]
-------------+------------------------------------------------
       asian |   .0304365   .0017131      .0270785    .0337944
--------------------------------------------------------------
(running mean on estimation sample)

Survey: Mean estimation

Number of strata =      1             Number of obs   = 10,000
Number of PSUs   = 10,000             Population size = 10,000
                                      Design df       =  9,999

--------------------------------------------------------------
             |             Linearized
             |       Mean   std. err.     [95% conf. interval]
-------------+------------------------------------------------
       black |     .12461   .0033444      .1180542    .1311658
--------------------------------------------------------------
(running mean on estimation sample)

Survey: Mean estimation

Number of strata =      1             Number of obs   = 10,000
```
© 2025 Pyrooz DC et al. *JAMA Network Open*.

```
Number of PSUs   = 10,000              Population size = 10,000
                                       Design df       =  9,999

--------------------------------------------------------------
             |             Linearized
             |       Mean   std. err.     [95% conf. interval]
-------------+------------------------------------------------
        hisp |    .160388   .0047813      .1510157    .1697603
--------------------------------------------------------------
(running mean on estimation sample)

Survey: Mean estimation

Number of strata =      1              Number of obs   = 10,000
Number of PSUs   = 10,000              Population size = 10,000
                                       Design df       =  9,999

--------------------------------------------------------------
             |             Linearized
             |       Mean   std. err.     [95% conf. interval]
-------------+------------------------------------------------
       white |     .62784   .0053894      .6172757    .6384043
--------------------------------------------------------------
(running mean on estimation sample)

Survey: Mean estimation

Number of strata =      1              Number of obs   = 10,000
Number of PSUs   = 10,000              Population size = 10,000
                                       Design df       =  9,999

--------------------------------------------------------------
             |             Linearized
             |       Mean   std. err.     [95% conf. interval]
-------------+------------------------------------------------
      other2 |   .0567255   .0022476      .0523198    .0611312
--------------------------------------------------------------
(running mean on estimation sample)

Survey: Mean estimation

Number of strata =      1              Number of obs   = 10,000
Number of PSUs   = 10,000              Population size = 10,000
                                       Design df       =  9,999

--------------------------------------------------------------
             |             Linearized
             |       Mean   std. err.     [95% conf. interval]
-------------+------------------------------------------------
         ed1 |   .0586871    .003106      .0525986    .0647755
--------------------------------------------------------------
(running mean on estimation sample)

Survey: Mean estimation

Number of strata =      1              Number of obs   = 10,000
Number of PSUs   = 10,000              Population size = 10,000
```

© 2025 Pyrooz DC et al. *JAMA Network Open*.

```
                                       Design df      =  9,999

--------------------------------------------------------------
             |             Linearized
             |       Mean   std. err.     [95% conf. interval]
-------------+------------------------------------------------
         ed2 |   .3252189   .0051961      .3150336    .3354043
--------------------------------------------------------------
(running mean on estimation sample)

Survey: Mean estimation

Number of strata =      1             Number of obs   = 10,000
Number of PSUs   = 10,000             Population size = 10,000
                                       Design df      =  9,999

--------------------------------------------------------------
             |             Linearized
             |       Mean   std. err.     [95% conf. interval]
-------------+------------------------------------------------
         ed3 |   .1856477   .0042119      .1773915    .1939039
--------------------------------------------------------------
(running mean on estimation sample)

Survey: Mean estimation

Number of strata =      1             Number of obs   = 10,000
Number of PSUs   = 10,000             Population size = 10,000
                                       Design df      =  9,999

--------------------------------------------------------------
             |             Linearized
             |       Mean   std. err.     [95% conf. interval]
-------------+------------------------------------------------
         ed4 |   .0929143   .0030666      .0869031    .0989255
--------------------------------------------------------------
(running mean on estimation sample)

Survey: Mean estimation

Number of strata =      1             Number of obs   = 10,000
Number of PSUs   = 10,000             Population size = 10,000
                                       Design df      =  9,999

--------------------------------------------------------------
             |             Linearized
             |       Mean   std. err.     [95% conf. interval]
-------------+------------------------------------------------
        inc1 |   .2313255    .004667      .2221772    .2404738
--------------------------------------------------------------
(running mean on estimation sample)

Survey: Mean estimation

Number of strata =      1             Number of obs   = 10,000
Number of PSUs   = 10,000             Population size = 10,000
                                       Design df      =  9,999
```
© 2025 Pyrooz DC et al. *JAMA Network Open*.

```
--------------------------------------------------------------
             |             Linearized
             |       Mean   std. err.     [95% conf. interval]
-------------+------------------------------------------------
        inc2 |   .1944878   .0043477      .1859654    .2030101
--------------------------------------------------------------
(running mean on estimation sample)

Survey: Mean estimation

Number of strata =      1             Number of obs   = 10,000
Number of PSUs   = 10,000             Population size = 10,000
                                      Design df       =  9,999

--------------------------------------------------------------
             |             Linearized
             |       Mean   std. err.     [95% conf. interval]
-------------+------------------------------------------------
        inc3 |   .2118374   .0044116      .2031898     .220485
--------------------------------------------------------------
(running mean on estimation sample)

Survey: Mean estimation

Number of strata =      1             Number of obs   = 10,000
Number of PSUs   = 10,000             Population size = 10,000
                                      Design df       =  9,999

--------------------------------------------------------------
             |             Linearized
             |       Mean   std. err.     [95% conf. interval]
-------------+------------------------------------------------
        inc4 |   .2141345   .0043923      .2055247    .2227442
--------------------------------------------------------------
(running mean on estimation sample)

Survey: Mean estimation

Number of strata =      1             Number of obs   = 10,000
Number of PSUs   = 10,000             Population size = 10,000
                                      Design df       =  9,999

--------------------------------------------------------------
             |             Linearized
             |       Mean   std. err.     [95% conf. interval]
-------------+------------------------------------------------
        inc5 |   .1482148   .0037251      .1409129    .1555167
--------------------------------------------------------------

.
. ***** Table 2: Prevalence of exposure to mass shootings
.
. summ    mass_8 pres_injure pres_when_year pres_local place_nhood place_bar
place_school place_shopping ///
>                 place_event place_college place_workplace place_worship place_movie
place_park place_othe
```
© 2025 Pyrooz DC et al. *JAMA Network Open*.

```
> r ///
>                  pres_media [aweight=weight]

    Variable |     Obs      Weight        Mean   Std. dev.       Min        Max
-------------+---------------------------------------------------------------
      mass_8 |  10,000       10000    .0694516   .2542332          0          1
pres_injured |  10,000       10000    .0217552   .1458906          0          1
pres_when_~r |     529  533.596418     2010.85   13.05436       1960       2023
  pres_local |  10,000       10000     .052889   .2238231          0          1
 place_nhood |  10,000       10000    .0240917   .1533417          0          1
-------------+---------------------------------------------------------------
   place_bar |  10,000       10000    .0085991   .0923362          0          1
place_school |  10,000       10000    .0083981     .09126          0          1
place_shop~g |  10,000       10000    .0079941   .0890563          0          1
 place_event |  10,000       10000    .0076778   .0872902          0          1
place_coll~e |  10,000       10000    .0048454   .0694436          0          1
-------------+---------------------------------------------------------------
place_work~e |  10,000       10000    .0021279   .0460824          0          1
place_wors~p |  10,000       10000    .0017971   .0423558          0          1
 place_movie |  10,000       10000    .0015469   .0393018          0          1
  place_park |  10,000       10000    .0015406   .0392227          0          1
 place_other |  10,000       10000    .0008329   .0288494          0          1
-------------+---------------------------------------------------------------
  pres_media |  10,000       10000    .0310332   .1734161          0          1

.
. foreach var of varlist mass_8 pres_injure {
  2.         svy: mean `var'
  3. }
(running mean on estimation sample)

Survey: Mean estimation

Number of strata =      1            Number of obs   = 10,000
Number of PSUs   = 10,000            Population size = 10,000
                                     Design df       =  9,999

--------------------------------------------------------------
             |             Linearized
             |       Mean   std. err.     [95% conf. interval]
-------------+------------------------------------------------
      mass_8 |   .0694516     .00283      .0639041     .074999
--------------------------------------------------------------
(running mean on estimation sample)

Survey: Mean estimation

Number of strata =      1            Number of obs   = 10,000
Number of PSUs   = 10,000            Population size = 10,000
                                     Design df       =  9,999

--------------------------------------------------------------
             |             Linearized
             |       Mean   std. err.     [95% conf. interval]
-------------+------------------------------------------------
pres_injured |   .0217552   .0016479       .018525    .0249855
--------------------------------------------------------------
```
© 2025 Pyrooz DC et al. *JAMA Network Open*.

```
.
. summ    mass_8 pres_injure pres_when_year pres_local place_nhood place_bar
place_school place_shopping ///
>                  place_event place_college place_workplace place_worship place_movie
place_park place_othe
> r ///
>                  pres_media [aweight=weight] if mass_8==1

    Variable |     Obs      Weight        Mean   Std. dev.       Min        Max
-------------+-----------------------------------------------------------------
      mass_8 |     696  694.515513           1          0          1          1
pres_injured |     696  694.515513    .3132433   .4641459          0          1
pres_when_~r |     529  533.596418     2010.85   13.05436       1960       2023
  pres_local |     696  694.515513    .7615236   .4264583          0          1
 place_nhood |     696  694.515513    .3468853   .4763212          0          1
-------------+-----------------------------------------------------------------
   place_bar |     696  694.515513     .123814   .3296061          0          1
place_school |     696  694.515513      .12092   .3262688          0          1
place_shop~g |     696  694.515513    .1151037   .3193766          0          1
 place_event |     696  694.515513    .1105485   .3137977          0          1
place_coll~e |     696  694.515513    .0697667   .2549366          0          1
-------------+-----------------------------------------------------------------
place_work~e |     696  694.515513    .0306386   .1724606          0          1
place_wors~p |     696  694.515513     .025875   .1588766          0          1
 place_movie |     696  694.515513    .0222726   .1476749          0          1
  place_park |     696  694.515513     .022183   .1473842          0          1
 place_other |     696  694.515513    .0119925   .1089301          0          1
-------------+-----------------------------------------------------------------
  pres_media |     696  694.515513    .4468321   .4975227          0          1

.
.
. ***** Table 3: Regression models predicting mass_8 and pres_injure
.
.
. logistic mass_8          i.generation2 i.male i.race_cat i.educ i.income
ib1.state_region i.newsint i.income_
> miss [pweight=weight], robust

Logistic regression                                     Number of obs = 10,000
                                                        Wald chi2(28) = 395.35
                                                        Prob > chi2   = 0.0000
Log pseudolikelihood = -2247.6191                       Pseudo R2     = 0.1089

------------------------------------------------------------------------------
             |               Robust
      mass_8 | Odds ratio   std. err.      z    P>|z|     [95% conf. interval]
-------------+----------------------------------------------------------------
 generation2 |
          1  |   .7478271   .0853301    -2.55   0.011     .5979649    .9352479
          2  |   .3119342   .0438407    -8.29   0.000     .2368269     .410861
          3  |   .1186624   .0218883   -11.56   0.000     .0826612     .170343
             |
      1.male |   1.564602   .1447116     4.84   0.000     1.305195    1.875566
             |
    race_cat |
```
© 2025 Pyrooz DC et al. *JAMA Network Open*.

```
          1  |   1.857602   .2134803     5.39   0.000     1.482963    2.326885
          2  |   1.024231   .1468748     0.17   0.867     .7732777    1.356626
          3  |   .3555017   .1109237    -3.31   0.001     .1928634    .6552901
          4  |   1.028472   .1849861     0.16   0.876     .7229216    1.463167
             |
     educyrs |
         12  |   .8697037   .2000702    -0.61   0.544     .5540608    1.365165
         13  |   .6364761   .1549276    -1.86   0.063      .394991    1.025598
         14  |   1.158047   .2944394     0.58   0.564     .7035642    1.906112
         16  |   1.006884   .2372492     0.03   0.977     .6344733    1.597885
         18  |   1.154412   .2883064     0.57   0.565     .7075861      1.8834
             |
      income |
2nd quint..  |   .9418365   .1543637    -0.37   0.715     .6830722    1.298627
3rd quint..  |   .8164809   .1305023    -1.27   0.205     .5968898    1.116858
4th quint..  |   .8782532   .1376659    -0.83   0.408     .6459429    1.194113
5th quint..  |   1.012622   .1666209     0.08   0.939     .7334786    1.398001
             |
state_region |
       west  |   1.390404   .2369964     1.93   0.053     .9955253    1.941912
    midwest  |   1.014418   .1512799     0.10   0.924     .7573168    1.358803
  northeast  |   1.021654   .1810624     0.12   0.904     .7218552    1.445966
         ca  |   2.135005   .3391269     4.78   0.000     1.563842    2.914773
         fl  |   1.070185   .2327927     0.31   0.755     .6987164    1.639144
         ny  |    2.11461   .3387764     4.67   0.000     1.544761    2.894671
         tx  |   1.287228   .2350963     1.38   0.167     .8998996    1.841268
             |
     newsint |
Only now ..  |   1.335053   .2378431     1.62   0.105     .9415745    1.892963
Some of t..  |   1.776477   .2891617     3.53   0.000     1.291241     2.44406
Most of t..  |   1.747624   .2941628     3.32   0.001     1.256529    2.430657
             |
1.income_m~s |   .4537347    .104094    -3.44   0.001     .2894161    .7113465
       _cons |   .0718422    .020916    -9.04   0.000     .0406034    .1271148
------------------------------------------------------------------------------
Note: _cons estimates baseline odds.

.         margins generation2 male race_cat

Predictive margins                                      Number of obs = 10,000
Model VCE: Robust

Expression: Pr(mass_8), predict()

------------------------------------------------------------------------------
             |            Delta-method
             |     Margin   std. err.      z    P>|z|     [95% conf. interval]
-------------+----------------------------------------------------------------
 generation2 |
          0  |   .1382416   .0106608    12.97   0.000     .1173468    .1591364
          1  |   .1081077   .0062198    17.38   0.000     .0959171    .1202983
          2  |   .0490928   .0047495    10.34   0.000     .0397839    .0584018
          3  |    .019468   .0028663     6.79   0.000     .0138501    .0250859
             |
        male |
          0  |   .0556008   .0034047    16.33   0.000     .0489278    .0622738
          1  |   .0824387   .0043263    19.06   0.000     .0739593    .0909181
```

© 2025 Pyrooz DC et al. *JAMA Network Open*.

```
              |
     race_cat |
            0  |    .0652345   .0034844    18.72   0.000     .0584052    .0720638
            1  |    .1104934   .0087358    12.65   0.000     .0933716    .1276153
            2  |    .0666272   .0075128     8.87   0.000     .0519023     .081352
            3  |    .0251387   .0073053     3.44   0.001     .0108205    .0394568
            4  |    .0668702   .0099271     6.74   0.000     .0474134     .086327
-------------------------------------------------------------------------------

.
. logistic pres_injure i.generation2 i.male i.race_cat i.educ i.income
ib1.state_region i.newsint i.income_miss
>  [pweight=weight], robust

Logistic regression                                     Number of obs = 10,000
                                                        Wald chi2(28) = 248.86
                                                        Prob > chi2   = 0.0000
Log pseudolikelihood = -870.32561                       Pseudo R2     = 0.1695

-------------------------------------------------------------------------------
              |               Robust
pres_injured | Odds ratio   std. err.      z    P>|z|     [95% conf. interval]
-------------+-----------------------------------------------------------------
 generation2 |
          1  |    .668886   .1236052    -2.18   0.030     .4656465    .9608328
          2  |   .1719514   .0446776    -6.78   0.000     .1033333    .2861349
          3  |     .01449   .0087447    -7.02   0.000     .0044399    .0472899
              |
      1.male |   1.889606   .3207556     3.75   0.000     1.354821    2.635484
              |
     race_cat |
          1  |   1.222109   .2512715     0.98   0.329     .8167679     1.82861
          2  |   .7897862   .1921449    -0.97   0.332     .4902562    1.272319
          3  |   .4294746   .2007763    -1.81   0.071     .1717935    1.073663
          4  |   .5561022    .197837    -1.65   0.099     .2769071    1.116799
              |
      educyrs |
         12  |    .769277   .2916016    -0.69   0.489     .3659543    1.617107
         13  |   .4989053   .2067511    -1.68   0.093     .2214482    1.123995
         14  |   .6299275   .2853405    -1.02   0.308       .25925    1.530602
         16  |   .8241989   .3256333    -0.49   0.625     .3799533    1.787861
         18  |   .9547129   .3996444    -0.11   0.912      .420299    2.168638
              |
       income |
2nd quint..  |   .8581358   .2529112    -0.52   0.604     .4816022    1.529057
3rd quint..  |   .9153184   .2510453    -0.32   0.747     .5347026    1.566867
4th quint..  |   1.001072   .2662779     0.00   0.997     .5943629    1.686082
5th quint..  |   1.004942   .2799734     0.02   0.986     .5821023    1.734934
              |
state_region |
        west  |   1.999119   .5824338     2.38   0.017     1.129393    3.538605
     midwest  |   .9472718     .27108    -0.19   0.850     .5406136    1.659825
   northeast  |    1.30586   .4163747     0.84   0.403      .699021    2.439512
          ca  |   2.885852   .8027817     3.81   0.000      1.67297    4.978058
          fl  |   .8753344   .3564038    -0.33   0.744     .3940908    1.944248
          ny  |   3.084612   .8379866     4.15   0.000     1.811163    5.253439
          tx  |   1.131718   .3761685     0.37   0.710      .589943    2.171034
```

© 2025 Pyrooz DC et al. *JAMA Network Open*.

```
             |
     newsint |
Only now ..  |   1.286573   .4649879     0.70   0.486     .6335734    2.612596
Some of t..  |   2.422093   .7685172     2.79   0.005     1.300496    4.510997
Most of t..  |   2.594182   .8391631     2.95   0.003     1.376115     4.89042
             |
1.income_m~s |   .3757283   .1609786    -2.28   0.022     .1622488    .8700939
       _cons |   .0203269   .0112084    -7.07   0.000     .0068979    .0599001
------------------------------------------------------------------------------
Note: _cons estimates baseline odds.

.         margins generation2 male race_cat

Predictive margins                                      Number of obs = 10,000
Model VCE: Robust

Expression: Pr(pres_injured), predict()

------------------------------------------------------------------------------
             |            Delta-method
             |     Margin   std. err.      z    P>|z|     [95% conf. interval]
-------------+----------------------------------------------------------------
 generation2 |
          0  |   .0562712   .0076275     7.38   0.000     .0413217    .0712208
          1  |   .0388062   .0038768    10.01   0.000     .0312078    .0464047
          2  |   .0104801   .0021473     4.88   0.000     .0062714    .0146887
          3  |   .0008979   .0005213     1.72   0.085    -.0001237    .0019196
             |
        male |
          0  |   .0149805    .001854     8.08   0.000     .0113467    .0186144
          1  |    .027278   .0025991    10.50   0.000     .0221839    .0323721
             |
    race_cat |
          0  |   .0234309   .0022605    10.37   0.000     .0190003    .0278615
          1  |    .028185   .0046139     6.11   0.000     .0191418    .0372281
          2  |   .0187941   .0038558     4.87   0.000     .0112368    .0263513
          3  |   .0105073   .0046014     2.28   0.022     .0014888    .0195259
          4  |   .0134709   .0043592     3.09   0.002     .0049272    .0220147
------------------------------------------------------------------------------

.
.
.
. ***** eTable 1: Descriptive statistics by location of mass shooting
.
. foreach x in place_nhood place_bar place_school place_shopping place_event {
  2.         tab generation [aweight=weight] if `x'==1
  3.         tab male [aweight=weight] if `x'==1
  4.         tab race_cat [aweight=weight] if `x'==1
  5.         tab educ [aweight=weight] if `x'==1
  6.         tab income [aweight=weight] if `x'==1
  7.         tab state_cat [aweight=weight] if `x'==1
  8.         tab newsint [aweight=weight] if `x'==1
  9. }

 generation |      Freq.     Percent        Cum.
------------+-----------------------------------
```

© 2025 Pyrooz DC et al. *JAMA Network Open*.

0	62.5393854	25.32	25.32
1	108.536814	43.94	69.26
2	47.1852151	19.10	88.36
3	28.0456311	11.35	99.72
4	.6929548406	0.28	100.00
Total	247	100.00	

male	Freq.	Percent	Cum.
0	98.3796211	39.83	39.83
1	148.620379	60.17	100.00
Total	247	100.00	

race_cat	Freq.	Percent	Cum.
0	118.461428	47.96	47.96
1	55.7485785	22.57	70.53
2	49.7194495	20.13	90.66
3	4.824987201	1.95	92.61
4	18.2455568	7.39	100.00
Total	247	100.00	

educyrs	Freq.	Percent	Cum.
10	31.3623649	12.70	12.70
12	78.5742494	31.81	44.51
13	26.7716383	10.84	55.35
14	24.3581259	9.86	65.21
16	54.0224594	21.87	87.08
18	31.91116218	12.92	100.00
Total	247	100.00	

income	Freq.	Percent	Cum.
lowest quintile	79.62630511	32.24	32.24
2nd quintile	41.8707192	16.95	49.19
3rd quintile	47.4282062	19.20	68.39
4th quintile	44.0178728	17.82	86.21
5th quintile	34.0568966	13.79	100.00
Total	247	100.00	

state_cat	Freq.	Percent	Cum.
0	134.850196	54.60	54.60
1	49.8946568	20.20	74.80
2	14.8135643	6.00	80.79
3	37.0825987	15.01	95.81
4	10.3589837	4.19	100.00
Total	247	100.00	

Political Interest	Freq.	Percent	Cum.

© 2025 Pyrooz DC et al. *JAMA Network Open*.

Hardly at all/Don't know	39.8053306	16.12	16.12
Only now and then	42.0827777	17.04	33.15
Some of the time	79.4325998	32.16	65.31
Most of the time	85.6792918	34.69	100.00
Total	247	100.00	

generation	Freq.	Percent	Cum.
0	22.7026619	25.80	25.80
1	47.5123056	53.99	79.79
2	13.1331893	14.92	94.71
3	4.6518432	5.29	100.00
Total	88	100.00	

male	Freq.	Percent	Cum.
0	26.99689701	30.68	30.68
1	61.00310299	69.32	100.00
Total	88	100.00	

race_cat	Freq.	Percent	Cum.
0	49.6883178	56.46	56.46
1	19.3446313	21.98	78.45
2	15.8128425	17.97	96.42
4	3.1542083	3.58	100.00
Total	88	100.00	

educyrs	Freq.	Percent	Cum.
10	4.29945907	4.89	4.89
12	30.3786468	34.52	39.41
13	9.79696018	11.13	50.54
14	10.0924403	11.47	62.01
16	24.5420499	27.89	89.90
18	8.89044374	10.10	100.00
Total	88	100.00	

income	Freq.	Percent	Cum.
lowest quintile	8.54445553	9.71	9.71
2nd quintile	19.1384024	21.75	31.46
3rd quintile	16.58232845	18.84	50.30
4th quintile	24.5837925	27.94	78.24
5th quintile	19.1510212	21.76	100.00
Total	88	100.00	

state_cat	Freq.	Percent	Cum.
0	41.051161	46.65	46.65

© 2025 Pyrooz DC et al. *JAMA Network Open*.

1	16.3787882	18.61	65.26
2	5.70725677	6.49	71.75
3	13.0621183	14.84	86.59
4	11.80067579	13.41	100.00
Total	88	100.00	

Political Interest	Freq.	Percent	Cum.
Hardly at all/Don't know	3.57187357	4.06	4.06
Only now and then	17.4407279	19.82	23.88
Some of the time	37.403886	42.50	66.38
Most of the time	29.5835125	33.62	100.00
Total	88	100.00	

generation	Freq.	Percent	Cum.
0	25.6014182	31.61	31.61
1	28.134975	34.73	66.34
2	19.6527891	24.26	90.60
3	6.929571113	8.56	99.16
4	.681246576	0.84	100.00
Total	81	100.00	

male	Freq.	Percent	Cum.
0	25.5205844	31.51	31.51
1	55.4794156	68.49	100.00
Total	81	100.00	

race_cat	Freq.	Percent	Cum.
0	41.0226956	50.65	50.65
1	18.8585665	23.28	73.93
2	18.1421197	22.40	96.33
4	2.97661821	3.67	100.00
Total	81	100.00	

educyrs	Freq.	Percent	Cum.
10	2.41368622	2.98	2.98
12	33.5585561	41.43	44.41
13	12.0731859	14.91	59.32
14	8.74884135	10.80	70.12
16	15.7872239	19.49	89.61
18	8.41850648	10.39	100.00
Total	81	100.00	

income	Freq.	Percent	Cum.
lowest quintile	21.7486953	26.85	26.85
2nd quintile	10.2155078	12.61	39.46

© 2025 Pyrooz DC et al. *JAMA Network Open*.

	Freq.	Percent	Cum.
3rd quintile	20.2409615	24.99	64.45
4th quintile	11.88482889	14.67	79.12
5th quintile	16.9100065	20.88	100.00
Total	81	100.00	

state_cat	Freq.	Percent	Cum.
0	41.3202466	51.01	51.01
1	13.8795065	17.14	68.15
2	2.75955766	3.41	71.55
3	14.7408388	18.20	89.75
4	8.29985039	10.25	100.00
Total	81	100.00	

Political Interest	Freq.	Percent	Cum.
Hardly at all/Don't know	7.06765922	8.73	8.73
Only now and then	13.899418	17.16	25.89
Some of the time	35.5932928	43.94	69.83
Most of the time	24.43963	30.17	100.00
Total	81	100.00	

generation	Freq.	Percent	Cum.
0	24.2805046	28.57	28.57
1	44.576147	52.44	81.01
2	10.7196736	12.61	93.62
3	5.42367474	6.38	100.00
Total	85	100.00	

male	Freq.	Percent	Cum.
0	34.28767399	40.34	40.34
1	50.71232601	59.66	100.00
Total	85	100.00	

race_cat	Freq.	Percent	Cum.
0	50.2544122	59.12	59.12
1	16.50644284	19.42	78.54
2	13.6048375	16.01	94.55
3	1.62397505	1.91	96.46
4	3.01033246	3.54	100.00
Total	85	100.00	

educyrs	Freq.	Percent	Cum.
10	2.90139028	3.41	3.41
12	15.67628904	18.44	21.86
13	13.9225755	16.38	38.24
14	14.1197891	16.61	54.85

© 2025 Pyrooz DC et al. *JAMA Network Open*.

16	28.15305277	33.12	87.97
18	10.2269034	12.03	100.00
Total	85	100.00	

income	Freq.	Percent	Cum.
lowest quintile	11.318194	13.32	13.32
2nd quintile	17.8929534	21.05	34.37
3rd quintile	14.04650118	16.53	50.89
4th quintile	21.9404362	25.81	76.70
5th quintile	19.80191521	23.30	100.00
Total	85	100.00	

state_cat	Freq.	Percent	Cum.
0	44.9370949	52.87	52.87
1	9.0205071	10.61	63.48
2	3.34749815	3.94	67.42
3	16.8361337	19.81	87.22
4	10.8587661	12.78	100.00
Total	85	100.00	

Political Interest	Freq.	Percent	Cum.
Hardly at all/Don't know	11.0470827	13.00	13.00
Only now and then	8.99136323	10.58	23.57
Some of the time	25.8448096	30.41	53.98
Most of the time	39.1167445	46.02	100.00
Total	85	100.00	

generation	Freq.	Percent	Cum.
0	25.6331929	33.73	33.73
1	32.25706869	42.44	76.17
2	12.1302291	15.96	92.13
3	5.97950934	7.87	100.00
Total	76	100.00	

male	Freq.	Percent	Cum.
0	31.3729889	41.28	41.28
1	44.6270111	58.72	100.00
Total	76	100.00	

race_cat	Freq.	Percent	Cum.
0	43.5519913	57.31	57.31
1	11.3401501	14.92	72.23
2	15.8857904	20.90	93.13
3	1.41318298	1.86	94.99
4	3.80888519	5.01	100.00

© 2025 Pyrooz DC et al. *JAMA Network Open*.

```
------------+-----------------------------------
      Total |         76      100.00

     educyrs |      Freq.     Percent        Cum.
-------------+-----------------------------------
          10 |  2.7803254        3.66        3.66
          12 | 20.1699715       26.54       30.20
          13 | 15.2595551       20.08       50.28
          14 | 11.5757935       15.23       65.51
          16 | 15.4583313       20.34       85.85
          18 | 10.7560231       14.15      100.00
-------------+-----------------------------------
       Total |         76      100.00

         income |      Freq.     Percent        Cum.
----------------+-----------------------------------
lowest quintile | 17.3833318       22.87       22.87
   2nd quintile | 10.0565361       13.23       36.11
   3rd quintile |  15.925748       20.95       57.06
   4th quintile | 17.7549257       23.36       80.42
   5th quintile |14.87945838       19.58      100.00
----------------+-----------------------------------
          Total |         76      100.00

  state_cat |      Freq.     Percent        Cum.
------------+-----------------------------------
          0 | 28.5684758       37.59       37.59
          1 | 27.7711272       36.54       74.13
          2 | 2.16615918        2.85       76.98
          3 | 6.20210535        8.16       85.14
          4 | 11.2921324       14.86      100.00
------------+-----------------------------------
      Total |         76      100.00

      Political Interest |      Freq.     Percent        Cum.
-------------------------+-----------------------------------
Hardly at all/Don't know | 10.5563104       13.89       13.89
       Only now and then | 10.8683205       14.30       28.19
        Some of the time | 28.4891539       37.49       65.68
        Most of the time | 26.0862151       34.32      100.00
-------------------------+-----------------------------------
                   Total |         76      100.00

. foreach x in generation male race_cat educ income state_cat newsint {
  2.         tab `x' [aweight=weight] if place_nhood==1
  3.         tab `x' [aweight=weight] if place_bar==1
  4.         tab `x' [aweight=weight] if place_school==1
  5.         tab `x' [aweight=weight] if place_shopping==1
  6.         tab `x' [aweight=weight] if place_event==1
  7.         tab `x' [aweight=weight] if place_college==1 | place_workplace==1 |
place_worship==1 | place_movie
> ==1 | place_park==1 | place_other==1
  8. }

 generation |      Freq.     Percent        Cum.
------------+-----------------------------------
          0 | 62.5393854       25.32       25.32
```

© 2025 Pyrooz DC et al. *JAMA Network Open*.

1	108.536814	43.94	69.26
2	47.1852151	19.10	88.36
3	28.0456311	11.35	99.72
4	.6929548406	0.28	100.00
Total	247	100.00	

generation	Freq.	Percent	Cum.
0	22.7026619	25.80	25.80
1	47.5123056	53.99	79.79
2	13.1331893	14.92	94.71
3	4.6518432	5.29	100.00
Total	88	100.00	

generation	Freq.	Percent	Cum.
0	25.6014182	31.61	31.61
1	28.134975	34.73	66.34
2	19.6527891	24.26	90.60
3	6.929571113	8.56	99.16
4	.681246576	0.84	100.00
Total	81	100.00	

generation	Freq.	Percent	Cum.
0	24.2805046	28.57	28.57
1	44.576147	52.44	81.01
2	10.7196736	12.61	93.62
3	5.42367474	6.38	100.00
Total	85	100.00	

generation	Freq.	Percent	Cum.
0	25.6331929	33.73	33.73
1	32.25706869	42.44	76.17
2	12.1302291	15.96	92.13
3	5.97950934	7.87	100.00
Total	76	100.00	

generation	Freq.	Percent	Cum.
0	36.40536819	30.59	30.59
1	53.093704	44.62	75.21
2	20.59141706	17.30	92.51
3	8.90951075	7.49	100.00
Total	119	100.00	

male	Freq.	Percent	Cum.
0	98.3796211	39.83	39.83
1	148.620379	60.17	100.00

© 2025 Pyrooz DC et al. *JAMA Network Open*.

Total	247	100.00	

male	Freq.	Percent	Cum.
0	26.99689701	30.68	30.68
1	61.00310299	69.32	100.00
Total	88	100.00	

male	Freq.	Percent	Cum.
0	25.5205844	31.51	31.51
1	55.4794156	68.49	100.00
Total	81	100.00	

male	Freq.	Percent	Cum.
0	34.28767399	40.34	40.34
1	50.71232601	59.66	100.00
Total	85	100.00	

male	Freq.	Percent	Cum.
0	31.3729889	41.28	41.28
1	44.6270111	58.72	100.00
Total	76	100.00	

male	Freq.	Percent	Cum.
0	50.5732192	42.50	42.50
1	68.4267808	57.50	100.00
Total	119	100.00	

race_cat	Freq.	Percent	Cum.
0	118.461428	47.96	47.96
1	55.7485785	22.57	70.53
2	49.7194495	20.13	90.66
3	4.824987201	1.95	92.61
4	18.2455568	7.39	100.00
Total	247	100.00	

race_cat	Freq.	Percent	Cum.
0	49.6883178	56.46	56.46
1	19.3446313	21.98	78.45
2	15.8128425	17.97	96.42
4	3.1542083	3.58	100.00
Total	88	100.00	

© 2025 Pyrooz DC et al. *JAMA Network Open*.

race_cat	Freq.	Percent	Cum.
0	41.0226956	50.65	50.65
1	18.8585665	23.28	73.93
2	18.1421197	22.40	96.33
4	2.97661821	3.67	100.00
Total	81	100.00	

race_cat	Freq.	Percent	Cum.
0	50.2544122	59.12	59.12
1	16.50644284	19.42	78.54
2	13.6048375	16.01	94.55
3	1.62397505	1.91	96.46
4	3.01033246	3.54	100.00
Total	85	100.00	

race_cat	Freq.	Percent	Cum.
0	43.5519913	57.31	57.31
1	11.3401501	14.92	72.23
2	15.8857904	20.90	93.13
3	1.41318298	1.86	94.99
4	3.80888519	5.01	100.00
Total	76	100.00	

race_cat	Freq.	Percent	Cum.
0	72.2314143	60.70	60.70
1	15.7845479	13.26	73.96
2	18.9425715	15.92	89.88
3	3.53469593	2.97	92.85
4	8.50677036	7.15	100.00
Total	119	100.00	

educyrs	Freq.	Percent	Cum.
10	31.3623649	12.70	12.70
12	78.5742494	31.81	44.51
13	26.7716383	10.84	55.35
14	24.3581259	9.86	65.21
16	54.0224594	21.87	87.08
18	31.91116218	12.92	100.00
Total	247	100.00	

educyrs	Freq.	Percent	Cum.
10	4.29945907	4.89	4.89
12	30.3786468	34.52	39.41
13	9.79696018	11.13	50.54
14	10.0924403	11.47	62.01
16	24.5420499	27.89	89.90

© 2025 Pyrooz DC et al. *JAMA Network Open*.

18	8.89044374	10.10	100.00
Total	88	100.00	

educyrs	Freq.	Percent	Cum.
10	2.41368622	2.98	2.98
12	33.5585561	41.43	44.41
13	12.0731859	14.91	59.32
14	8.74884135	10.80	70.12
16	15.7872239	19.49	89.61
18	8.41850648	10.39	100.00
Total	81	100.00	

educyrs	Freq.	Percent	Cum.
10	2.90139028	3.41	3.41
12	15.67628904	18.44	21.86
13	13.9225755	16.38	38.24
14	14.1197891	16.61	54.85
16	28.15305277	33.12	87.97
18	10.2269034	12.03	100.00
Total	85	100.00	

educyrs	Freq.	Percent	Cum.
10	2.7803254	3.66	3.66
12	20.1699715	26.54	30.20
13	15.2595551	20.08	50.28
14	11.5757935	15.23	65.51
16	15.4583313	20.34	85.85
18	10.7560231	14.15	100.00
Total	76	100.00	

educyrs	Freq.	Percent	Cum.
10	3.05327361	2.57	2.57
12	26.2834534	22.09	24.65
13	23.4311099	19.69	44.34
14	11.3809217	9.56	53.91
16	33.89534824	28.48	82.39
18	20.9558932	17.61	100.00
Total	119	100.00	

income	Freq.	Percent	Cum.
lowest quintile	79.62630511	32.24	32.24
2nd quintile	41.8707192	16.95	49.19
3rd quintile	47.4282062	19.20	68.39
4th quintile	44.0178728	17.82	86.21
5th quintile	34.0568966	13.79	100.00
Total	247	100.00	

© 2025 Pyrooz DC et al. *JAMA Network Open*.

income	Freq.	Percent	Cum.
lowest quintile	8.54445553	9.71	9.71
2nd quintile	19.1384024	21.75	31.46
3rd quintile	16.58232845	18.84	50.30
4th quintile	24.5837925	27.94	78.24
5th quintile	19.1510212	21.76	100.00
Total	88	100.00	

income	Freq.	Percent	Cum.
lowest quintile	21.7486953	26.85	26.85
2nd quintile	10.2155078	12.61	39.46
3rd quintile	20.2409615	24.99	64.45
4th quintile	11.88482889	14.67	79.12
5th quintile	16.9100065	20.88	100.00
Total	81	100.00	

income	Freq.	Percent	Cum.
lowest quintile	11.318194	13.32	13.32
2nd quintile	17.8929534	21.05	34.37
3rd quintile	14.04650118	16.53	50.89
4th quintile	21.9404362	25.81	76.70
5th quintile	19.80191521	23.30	100.00
Total	85	100.00	

income	Freq.	Percent	Cum.
lowest quintile	17.3833318	22.87	22.87
2nd quintile	10.0565361	13.23	36.11
3rd quintile	15.925748	20.95	57.06
4th quintile	17.7549257	23.36	80.42
5th quintile	14.87945838	19.58	100.00
Total	76	100.00	

income	Freq.	Percent	Cum.
lowest quintile	22.5777683	18.97	18.97
2nd quintile	23.0932703	19.41	38.38
3rd quintile	15.0840602	12.68	51.05
4th quintile	31.8638339	26.78	77.83
5th quintile	26.3810673	22.17	100.00
Total	119	100.00	

state_cat	Freq.	Percent	Cum.
0	134.850196	54.60	54.60
1	49.8946568	20.20	74.80
2	14.8135643	6.00	80.79
3	37.0825987	15.01	95.81

© 2025 Pyrooz DC et al. *JAMA Network Open*.

4	10.3589837	4.19	100.00
Total	247	100.00	

state_cat	Freq.	Percent	Cum.
0	41.051161	46.65	46.65
1	16.3787882	18.61	65.26
2	5.70725677	6.49	71.75
3	13.0621183	14.84	86.59
4	11.80067579	13.41	100.00
Total	88	100.00	

state_cat	Freq.	Percent	Cum.
0	41.3202466	51.01	51.01
1	13.8795065	17.14	68.15
2	2.75955766	3.41	71.55
3	14.7408388	18.20	89.75
4	8.29985039	10.25	100.00
Total	81	100.00	

state_cat	Freq.	Percent	Cum.
0	44.9370949	52.87	52.87
1	9.0205071	10.61	63.48
2	3.34749815	3.94	67.42
3	16.8361337	19.81	87.22
4	10.8587661	12.78	100.00
Total	85	100.00	

state_cat	Freq.	Percent	Cum.
0	28.5684758	37.59	37.59
1	27.7711272	36.54	74.13
2	2.16615918	2.85	76.98
3	6.20210535	8.16	85.14
4	11.2921324	14.86	100.00
Total	76	100.00	

state_cat	Freq.	Percent	Cum.
0	66.4773159	55.86	55.86
1	14.9491851	12.56	68.43
2	7.48967087	6.29	74.72
3	16.4878564	13.86	88.57
4	13.59597166	11.43	100.00
Total	119	100.00	

Political Interest	Freq.	Percent	Cum.
Hardly at all/Don't know	39.8053306	16.12	16.12

© 2025 Pyrooz DC et al. *JAMA Network Open*.

```
       Only now and then | 42.0827777       17.04       33.15
        Some of the time | 79.4325998       32.16       65.31
        Most of the time | 85.6792918       34.69      100.00
-------------------------+-----------------------------------
                   Total |        247      100.00

      Political Interest |      Freq.     Percent        Cum.
-------------------------+-----------------------------------
Hardly at all/Don't know | 3.57187357        4.06        4.06
       Only now and then | 17.4407279       19.82       23.88
        Some of the time |  37.403886       42.50       66.38
        Most of the time | 29.5835125       33.62      100.00
-------------------------+-----------------------------------
                   Total |         88      100.00

      Political Interest |      Freq.     Percent        Cum.
-------------------------+-----------------------------------
Hardly at all/Don't know | 7.06765922        8.73        8.73
       Only now and then |  13.899418       17.16       25.89
        Some of the time | 35.5932928       43.94       69.83
        Most of the time |   24.43963       30.17      100.00
-------------------------+-----------------------------------
                   Total |         81      100.00

      Political Interest |      Freq.     Percent        Cum.
-------------------------+-----------------------------------
Hardly at all/Don't know | 11.0470827       13.00       13.00
       Only now and then | 8.99136323       10.58       23.57
        Some of the time | 25.8448096       30.41       53.98
        Most of the time | 39.1167445       46.02      100.00
-------------------------+-----------------------------------
                   Total |         85      100.00

      Political Interest |      Freq.     Percent        Cum.
-------------------------+-----------------------------------
Hardly at all/Don't know | 10.5563104       13.89       13.89
       Only now and then | 10.8683205       14.30       28.19
        Some of the time | 28.4891539       37.49       65.68
        Most of the time | 26.0862151       34.32      100.00
-------------------------+-----------------------------------
                   Total |         76      100.00

      Political Interest |      Freq.     Percent        Cum.
-------------------------+-----------------------------------
Hardly at all/Don't know | 9.89267417        8.31        8.31
       Only now and then | 10.8188701        9.09       17.40
        Some of the time | 51.7498161       43.49       60.89
        Most of the time | 46.5386396       39.11      100.00
-------------------------+-----------------------------------
                   Total |        119      100.00

.
.
. ***** eTable 2: Descriptive statistics by media coverage
.
. foreach x in generation male race_cat educ income state_cat newsint {
  2.          tab `x' [aweight=weight] if pres_media == 1 & mass_8==1
```

© 2025 Pyrooz DC et al. *JAMA Network Open*.

```
  3.         tab `x' [aweight=weight] if pres_media == 0 & mass_8==1
  4. }

 generation |      Freq.     Percent        Cum.
------------+-----------------------------------
          0 |  95.819315       29.85       29.85
          1 | 161.591451       50.34       80.19
          2 | 40.0298759       12.47       92.66
          3 |22.12963615        6.89       99.55
          4 | 1.42972223        0.45      100.00
------------+-----------------------------------
      Total |        321      100.00

 generation |      Freq.     Percent        Cum.
------------+-----------------------------------
          0 | 102.146254       27.24       27.24
          1 |152.6349061       40.70       67.94
          2 | 82.8010506       22.08       90.02
          3 | 37.4177896        9.98      100.00
------------+-----------------------------------
      Total |        375      100.00

       male |      Freq.     Percent        Cum.
------------+-----------------------------------
          0 |124.6912126       38.84       38.84
          1 |196.3087874       61.16      100.00
------------+-----------------------------------
      Total |        321      100.00

       male |      Freq.     Percent        Cum.
------------+-----------------------------------
          0 | 142.631855       38.04       38.04
          1 | 232.368145       61.96      100.00
------------+-----------------------------------
      Total |        375      100.00

   race_cat |      Freq.     Percent        Cum.
------------+-----------------------------------
          0 | 195.480836       60.90       60.90
          1 | 55.6692644       17.34       78.24
          2 | 53.0371042       16.52       94.76
          3 |6.538320926        2.04       96.80
          4 | 10.2744743        3.20      100.00
------------+-----------------------------------
      Total |        321      100.00

   race_cat |      Freq.     Percent        Cum.
------------+-----------------------------------
          0 |181.4938514       48.40       48.40
          1 |  80.845872       21.56       69.96
          2 | 78.6020052       20.96       90.92
          3 | 4.98754659        1.33       92.25
          4 | 29.0707249        7.75      100.00
------------+-----------------------------------
      Total |        375      100.00

    educyrs |      Freq.     Percent        Cum.
```
© 2025 Pyrooz DC et al. *JAMA Network Open*.

10	15.5522811	4.84	4.84
12	81.0810145	25.26	30.10
13	51.4493233	16.03	46.13
14	26.75941282	8.34	54.47
16	92.0448301	28.67	83.14
18	54.1131382	16.86	100.00
Total	321	100.00	

educyrs	Freq.	Percent	Cum.
10	30.309784	8.08	8.08
12	122.872031	32.77	40.85
13	50.7193724	13.53	54.37
14	52.6426849	14.04	68.41
16	80.3306931	21.42	89.83
18	38.1254349	10.17	100.00
Total	375	100.00	

income	Freq.	Percent	Cum.
lowest quintile	60.8838033	18.97	18.97
2nd quintile	45.143884	14.06	33.03
3rd quintile	59.7342832	18.61	51.64
4th quintile	87.0169885	27.11	78.75
5th quintile	68.221041	21.25	100.00
Total	321	100.00	

income	Freq.	Percent	Cum.
lowest quintile	99.549378	26.55	26.55
2nd quintile	76.2372173	20.33	46.88
3rd quintile	69.3870823	18.50	65.38
4th quintile	66.0875301	17.62	83.00
5th quintile	63.7387923	17.00	100.00
Total	375	100.00	

state_cat	Freq.	Percent	Cum.
0	162.997119	50.78	50.78
1	52.5353441	16.37	67.14
2	16.4845466	5.14	72.28
3	53.2995703	16.60	88.88
4	35.6834202	11.12	100.00
Total	321	100.00	

state_cat	Freq.	Percent	Cum.
0	194.16719	51.78	51.78
1	78.8228162	21.02	72.80
2	19.7894474	5.28	78.07
3	51.1196695	13.63	91.71

© 2025 Pyrooz DC et al. *JAMA Network Open*.

```
          4 | 31.1008764        8.29      100.00
------------+-----------------------------------
      Total |        375      100.00

      Political Interest |      Freq.     Percent        Cum.
-------------------------+-----------------------------------
Hardly at all/Don't know | 18.1501276        5.65        5.65
       Only now and then | 38.8175528       12.09       17.75
        Some of the time | 125.948736       39.24       56.98
        Most of the time | 138.083584       43.02      100.00
-------------------------+-----------------------------------
                   Total |        321      100.00

      Political Interest |      Freq.     Percent        Cum.
-------------------------+-----------------------------------
Hardly at all/Don't know | 62.1775023       16.58       16.58
       Only now and then | 64.3695531       17.17       33.75
        Some of the time |134.1570515       35.78       69.52
        Most of the time | 114.295893       30.48      100.00
-------------------------+-----------------------------------
                   Total |        375      100.00

.
. foreach x in pres_injure pres_local place_nhood place_bar place_school
place_shopping ///
>                 place_event {
  2.         tab `x' [aweight=weight] if pres_media == 1 & mass_8==1
  3.         tab `x' [aweight=weight] if pres_media == 0 & mass_8==1
  4. }

  Present - |
   Were you |
 physically |
    injured |      Freq.     Percent        Cum.
------------+-----------------------------------
         no | 181.611445       56.58       56.58
        yes | 139.388555       43.42      100.00
------------+-----------------------------------
      Total |        321      100.00

  Present - |
   Were you |
 physically |
    injured |      Freq.     Percent        Cum.
------------+-----------------------------------
         no | 294.182974       78.45       78.45
        yes | 80.8170261       21.55      100.00
------------+-----------------------------------
      Total |        375      100.00

  Present - |
      Local |
  community |      Freq.     Percent        Cum.
------------+-----------------------------------
         no | 63.0505631       19.64       19.64
        yes | 257.949437       80.36      100.00
------------+-----------------------------------
```

© 2025 Pyrooz DC et al. *JAMA Network Open*.

```
      Total |        321      100.00

  Present - |
      Local |
  community |      Freq.     Percent        Cum.
------------+-----------------------------------
         no | 102.168329       27.24       27.24
        yes | 272.831671       72.76      100.00
------------+-----------------------------------
      Total |        375      100.00

pres_place= |
=Neighborho |
         od |      Freq.     Percent        Cum.
------------+-----------------------------------
          0 |239.8157675       74.71       74.71
          1 | 81.1842325       25.29      100.00
------------+-----------------------------------
      Total |        321      100.00

pres_place= |
=Neighborho |
         od |      Freq.     Percent        Cum.
------------+-----------------------------------
          0 | 216.451724       57.72       57.72
          1 | 158.548276       42.28      100.00
------------+-----------------------------------
      Total |        375      100.00

pres_place= |
    =Bar or |
 restaurant |      Freq.     Percent        Cum.
------------+-----------------------------------
          0 |  268.18634       83.55       83.55
          1 | 52.8136598       16.45      100.00
------------+-----------------------------------
      Total |        321      100.00

pres_place= |
    =Bar or |
 restaurant |      Freq.     Percent        Cum.
------------+-----------------------------------
          0 | 340.902754       90.91       90.91
          1 | 34.0972463        9.09      100.00
------------+-----------------------------------
      Total |        375      100.00

pres_place= |
    =School |      Freq.     Percent        Cum.
------------+-----------------------------------
          0 |278.2320802       86.68       86.68
          1 | 42.7679198       13.32      100.00
------------+-----------------------------------
      Total |        321      100.00

pres_place= |
    =School |      Freq.     Percent        Cum.
```

© 2025 Pyrooz DC et al. *JAMA Network Open*.

```
------------+-----------------------------------
          0 | 333.384877       88.90       88.90
          1 | 41.6151233       11.10      100.00
------------+-----------------------------------
      Total |        375      100.00

pres_place= |
  =Shopping |
    mall or |
    grocery |
      store |      Freq.     Percent        Cum.
------------+-----------------------------------
          0 | 282.697884       88.07       88.07
          1 | 38.3021164       11.93      100.00
------------+-----------------------------------
      Total |        321      100.00

pres_place= |
  =Shopping |
    mall or |
    grocery |
      store |      Freq.     Percent        Cum.
------------+-----------------------------------
          0 | 333.113644       88.83       88.83
          1 | 41.8863559       11.17      100.00
------------+-----------------------------------
      Total |        375      100.00

pres_place= |
   =Outdoor |
 concert or |
      event |      Freq.     Percent        Cum.
------------+-----------------------------------
          0 | 296.801662       92.46       92.46
          1 | 24.1983378        7.54      100.00
------------+-----------------------------------
      Total |        321      100.00

pres_place= |
   =Outdoor |
 concert or |
      event |      Freq.     Percent        Cum.
------------+-----------------------------------
          0 | 322.892583       86.10       86.10
          1 | 52.1074172       13.90      100.00
------------+-----------------------------------
      Total |        375      100.00

.
. tabstat pres_when_year [aweight=weight] if mass_8==1, by(pres_media)

Summary for variables: pres_when_year
Group variable: pres_media (Present - Incident covered widely by news media)

pres_media |      Mean
-----------+----------
        no |  2009.518
```
© 2025 Pyrooz DC et al. *JAMA Network Open*.

```
       yes |  2012.459
-----------+----------
     Total |   2010.85
----------------------

.
. ***** Reviewer request for information by decade
. gen decade50s = 0

.         replace decade50s = 1 if birthyr<1960
(2,271 real changes made)

. gen decade60s = 0

.         replace decade60s = 1 if
inlist(birthyr,1960,1961,1962,1963,1964,1965,1966,1967,1968,1969)
(1,793 real changes made)

. gen decade70s = 0

.         replace decade70s = 1 if
inlist(birthyr,1970,1971,1972,1973,1974,1975,1976,1977,1978,1979)
(1,443 real changes made)

. gen decade80s = 0

.         replace decade80s = 1 if
inlist(birthyr,1980,1981,1982,1983,1984,1985,1986,1987,1988,1989)
(1,729 real changes made)

. gen decade90s = 0

.         replace decade90s = 1 if
inlist(birthyr,1990,1991,1992,1993,1994,1995,1996,1997,1998,1999)
(1,874 real changes made)

. gen decade00s = 0

.         replace decade00s = 1 if birthyr>=2000
(890 real changes made)

. gen decade = "1950s or earlier" if decade50s==1
(7,729 missing values generated)

.         replace decade = "1960s" if decade60s==1
(1,793 real changes made)

.         replace decade = "1970s" if decade70s==1
(1,443 real changes made)

.         replace decade = "1980s" if decade80s==1
(1,729 real changes made)

.         replace decade = "1990s" if decade90s==1
(1,874 real changes made)

.         replace decade = "2000s" if decade00s==1
```
© 2025 Pyrooz DC et al. *JAMA Network Open*.

```
(890 real changes made)

.
. tab  decade mass_8 [aweight=weight]

                 | Present - Personally
                 |    been physically
                 | present on the scene
                 |  of a mass shooting
          decade |        no        yes |     Total
-----------------+----------------------+----------
1950s or earlier | 2,258.731 31.1851274 | 2,289.916
           1960s | 1,734.075  55.571159 |1,789.6459
           1970s |  1,385.28  80.042627 | 1,465.322
           1980s | 1,557.115  169.21524 |  1,726.33
           1990s | 1,541.866  226.68869 | 1,768.555
           2000s | 828.41778  131.81266 | 960.23044
-----------------+----------------------+----------
           Total | 9,305.484  694.51551 |    10,000

. tab  decade pres_injure [aweight=weight]

                 |  Present - Were you
                 |  physically injured
          decade |        no        yes |     Total
-----------------+----------------------+----------
1950s or earlier |  2,288.86  1.0563659 | 2,289.916
           1960s | 1,782.729   6.916737 |1,789.6459
           1970s |   1,449.6  15.722272 | 1,465.322
           1980s | 1,669.729  56.601314 |  1,726.33
           1990s | 1,690.161   78.39427 | 1,768.555
           2000s | 901.36909  58.861347 | 960.23044
-----------------+----------------------+----------
           Total | 9,782.448  217.55231 |    10,000

.
. log close
      name:  <unnamed>
       log:  /XXXXX/YouGov Survey/Exposure to MV/Analysis/log.smcl
  log type:  smcl
 closed on:  25 Jan 2025, 12:49:12
---------------------------------------------------------------------------------------
```
