# Supplementary material for: Direct Exposure to Mass Shootings Among US Adults
Source: JAMA Netw Open. 2025 Mar 7;8(3):e250283. doi: 10.1001/jamanetworkopen.2025.0283 (PMC11889466; doi:10.1001/jamanetworkopen.2025.0283)
Supplement: Supplement 2. — Data Sharing Statement [file jamanetwopen-e250283-s002.pdf]

Data Sharing Statement

Pyrooz. Direct Exposure to Mass Shootings Among US Adults. *JAMA Netw Open*. Published March 07, 2025. doi:10.1001/jamanetworkopen.2025.0283

Data
Data available: Yes
Data types: Deidentified participant data
How to access data: The data will be archived in an online and publicly accessible repository: https://osf.io/7492y/?view_only=24b5908f121246eb9becad3ced7751f6
When available: With publication

Supporting Documents
Document types: Statistical/analytic code
How to access documents: The code will be archived in an online and publicly accessible repository: https://osf.io/7492y/?view_only=24b5908f121246eb9becad3ced7751f6
When available: With publication

Additional Information
Who can access the data: All members of the public
Types of analyses: For any purpose
Mechanisms of data availability: The data will be archived in an online and publicly accessible repository
